# Cellular Senescence and Extracellular Vesicles in the Pathogenesis and Treatment of Obesity—A Narrative Review

**DOI:** 10.3390/ijms25147943

**Published:** 2024-07-20

**Authors:** Yicong Liang, Devesh Kaushal, Robert Beaumont Wilson

**Affiliations:** 1 Bankstown Hospital, University of New South Wales, Sydney, NSW 2560, Australia; z5083614@zmail.unsw.edu.au; 2 Campbelltown Hospital, Western Sydney University, Sydney, NSW 2560, Australia; d.kaushal@westernsydney.edu.au; 3 School of Clinical Medicine, University of New South Wales, High St., Kensington, Sydney, NSW 2052, Australia

**Keywords:** adiponectin, aging, AMPK, autophagy, cellular senescence, DDR, ER stress, epigenome, exosomes, extracellular vesicles, hypertrophic obesity, insulin resistance, lipotoxicity, metabolic syndrome, miRNA, NEFA, obesogenic environment, p53, ROS, SASP, senolytic, SIRT-1, VAT, Western-type diet

## Abstract

This narrative review explores the pathophysiology of obesity, cellular senescence, and exosome release. When exposed to excessive nutrients, adipocytes develop mitochondrial dysfunction and generate reactive oxygen species with DNA damage. This triggers adipocyte hypertrophy and hypoxia, inhibition of adiponectin secretion and adipogenesis, increased endoplasmic reticulum stress and maladaptive unfolded protein response, metaflammation, and polarization of macrophages. Such feed-forward cycles are not resolved by antioxidant systems, heat shock response pathways, or DNA repair mechanisms, resulting in transmissible cellular senescence via autocrine, paracrine, and endocrine signaling. Senescence can thus affect preadipocytes, mature adipocytes, tissue macrophages and lymphocytes, hepatocytes, vascular endothelium, pancreatic β cells, myocytes, hypothalamic nuclei, and renal podocytes. The senescence-associated secretory phenotype is closely related to visceral adipose tissue expansion and metaflammation; inhibition of SIRT-1, adiponectin, and autophagy; and increased release of exosomes, exosomal micro-RNAs, pro-inflammatory adipokines, and saturated free fatty acids. The resulting hypernefemia, insulin resistance, and diminished fatty acid β-oxidation lead to lipotoxicity and progressive obesity, metabolic syndrome, and physical and cognitive functional decline. Weight cycling is related to continuing immunosenescence and exposure to palmitate. Cellular senescence, exosome release, and the transmissible senescence-associated secretory phenotype contribute to obesity and metabolic syndrome. Targeted therapies have interrelated and synergistic effects on cellular senescence, obesity, and premature aging.

## 1. Introduction

Obesity is defined as ‘abnormal and excessive fat accumulation that may impair health’ and was classified as a chronic, relapsing, progressive disease by the World Health Organization in 1997 [1]. A body mass index (BMI) of 25–29.9 kg/m^2^ in Caucasian people or 23–24.9 kg/m^2^ in Asian people is defined as overweight, while obesity in Caucasians is defined as BMI ≥ 30.0 kg/m^2^ or ≥25.0 kg/m^2^ in Asians [2]. The current global epidemic of obesity is related to increased urbanization, sedentary lifestyles, aging populations, and widespread availability and consumption of highly processed, energy-dense food and sugar-sweetened beverages (*Western dietary pattern*) [1]. Food, social, and built environments that are obesogenic lead to an imbalance in energy intake and energy expenditure and result in the storage of excess energy as fat by adipocyte hypertrophy. This involves epigenetic alterations in the expression of genes that regulate cellular metabolism and proliferation, proteostasis, inflammation, and thermogenesis. Sustained weight loss from conventional lifestyle interventions is usually unsuccessful due to food reward pathways, feedback control of energy intake, and modeling based on the *constrained energy expenditure hypothesis*, as well as existing comorbidities and behavioral patterns. Multiple controlled feeding studies in humans comparing isocaloric, protein-equivalent low-fat or low-carbohydrate diets have shown similar effects on short-term weight loss. However, the composition of diets may influence the pattern of fat deposition, with overconsumption of refined simple sugars (fructose, sucrose) greatly contributing to fat deposition in the liver and trans fats and saturated fats in visceral adipose tissue (VAT) [3].

Compared with metabolically healthy individuals with normal weight (MHNW), individuals with obesity have an increased risk of multiple co-morbidities, including type II diabetes mellitus (T2DM), non-alcoholic steatohepatitis (NASH), hypertension, dyslipidemia, accelerated atherosclerosis, hypogonadism or polycystic ovary syndrome, obesity-associated cancer, cholelithiasis, osteoarthritis, obstructive sleep apnea (OSA), chronic kidney disease (CKD), Alzheimer’s disease, accelerated epigenetic aging, and premature death [4,5]. For example, each 5 kg/m^2^ increase in BMI above 25 kg/m^2^ is associated with a 30% higher mortality [6]. Patients with the same chronological age can have a divergent biological age, which is related to environmental exposures, diet, lifestyle, physical activity, metabolic fitness, and DNA methylation pattern—the *epigenetic clock*. Epigenetic aging can be measured by differentially methylated regions (DMRs) in CpG islands of longevity genes, with overlap between BMI and metabolic alterations in *MTOR*, *ULK1*, *ADCY6*, *IGF1R*, *CREB5,* and *RELA* genes. Of these, *CREB5*, *RELA*, and *ULK1* DMRs were statistically correlated with age [6]. 

BMI measurements may be inaccurate in predicting metabolic risk due to differences in body morphology, demography, ethnicity, and VAT volume [7,8,9]. In 2023, the US AMA recommended the use of other validated obesity criteria apart from BMI, including visceral fat volume, body adiposity index, body composition, relative fat mass, waist circumference (WC), and genetic/metabolic factors [6]. This is because increased VAT volume is an important factor in the genesis of insulin resistance and metabolic syndrome, and a proportion of “normal” weight individuals (BMI 18.5–24.9 kg/m^2^) will have expanded VAT volume and be metabolically unhealthy (MUNW). This is reflected in the BMI and waist circumference criteria being adjusted for ethnicity, as persons with Asian ethnicity develop VAT expansion, central obesity, diabetes, and metabolic syndrome at a lower BMI and WC than Caucasians [7,8,9]. Premenopausal women are somewhat protected from VAT expansion by ovarian estrogen secretion and gynoid pattern fat deposition, and they can have an obese or overweight BMI but be metabolically healthy (MHO). Menopause is associated with ovarian senescence and changes in body composition and adipokine production, with post-menopausal women developing a more android phenotype associated with central obesity, myosteatosis, loss of lean muscle, VAT expansion, and insulin resistance [5,10].

The following narrative review describes a unifying hypothesis for the pathophysiology of energy-dense diets, insulin resistance, oxidative stress, and impairment of mitochondrial function during the development of obesity. This involves adipocyte hypertrophy and VAT expansion, lipotoxicity, chronic metabolic inflammation (metaflammation), cellular senescence, exosome release, and epigenetic control of gene expression. Senolytic therapy to remove accumulated senescent cells and restore replicative capacity has been used in progeroid animals or aged (twenty-four-month-old) C57BL/6 mice, as well as in hypertrophic obesity models [11]. The effect of senotherapy and anti-obesity treatments on stress-induced cellular senescence, exosome miRNA, adipokines, and epigenetic changes in obesity and metabolic syndrome is reviewed. 

## 2. Methods

A literature search of PubMed, MEDLINE, and Cochrane Library databases to 30 June 2024 was conducted using MeSH keyword terms “obesity” AND “metabolic syndrome” in combination with “Western diet”, “adipocyte hypertrophy”, “adiponectin”, “aging”, “senescence”, “exosomes”, “extracellular vesicles”, “microRNA”, “lipotoxicity”, “type 2 diabetes mellitus”, “non-alcoholic fatty liver disease”, “DASH diet”, “Mediterranean diet”, “intermittent fasting”, “bariatric surgery”, “senotherapeutic agents”, OR “glucagon-like peptide-1 receptor agonist”. Search results were limited to English language. The titles and abstracts of the search results were screened. The full manuscripts that may be relevant were read. The reference lists for the papers were examined for potential studies to be included in this review.

## 3. Apoptosis versus Senescence

Apoptosis is a normal phenomenon of programmed cell death during development, growth, and aging [12,13,14,15,16]. Physiological apoptosis plays an important role in regulating the cell population and acting as a defense mechanism against immune reaction, genotoxicity, or cell damage by toxins or disease. Insufficient or excessive apoptosis can lead to cancer, obesity, neurodegenerative or autoimmune diseases. Cellular senescence refers to a state of irreversible cell cycle arrest whereby cells cease to replicate but do not undergo apoptosis [15] due to the activation of senescent cell anti-apoptotic pathways (SCAPs). Such resistance to both intrinsic (mitochondrial-mediated) and extrinsic (death receptor-mediated) apoptosis is mediated by increased expression of the anti-apoptotic members of the B-cell lymphoma 2 (Bcl-2) family proteins. This limits the mitochondrial outer membrane permeability (MOMP) and prevents the release of mitochondrial cytochrome c, which otherwise would activate apoptotic protease activating factor-1 (APAF1) and caspase-9 [16]. Other SCAPs involve Src kinase, PI3K/AKT/mTOR, heat shock protein 90 (HSP90), or p53/serpines, all of which are involved in cellular senescence in obesity [17]. 

## 4. Obesity and Stress-Induced Cellular Senescence 

Cellular senescence was first described in 1965 in human diploid fibroblasts [18]. These cells remained alive in vitro whilst in a non-dividing state after 50 replication cycles—the *Hayflick limit*. Cellular senescence can be classified as replicative, ultraviolet/γ-radiation/chemotherapy, oncogene (Harvey rat sarcoma viral oncogene homolog (HRAS^G12V^)), or stress-induced senescence. Stress-induced senescence is related to genotoxic or oxidative stress involving the oxidant effects of reactive oxygen species (ROS) and is a feature of hypertrophic obesity [18]. Mitochondrial dysfunction-associated senescence (MiDAS) is caused by dysfunctional mitochondria producing reactive oxygen species (ROS) due to electron leakage from the electron transport chain onto molecular oxygen [16]. During respiration, between 0.2 and 2% of the molecular oxygen used by mitochondria is converted to superoxides, and over 90% of the ROS generated in the body is by mitochondria [19].

Hyperglycemia and incomplete or excessive mitochondrial β-oxidation of free fatty acids results in increased generation of mitochondrial superoxides. Mitochondrial oxidative stress can be exacerbated by a deficiency of the mitochondrial antioxidant enzyme superoxide dismutase 2 (SOD2) [16]. SOD2 normally converts superoxide anions (O_2_^•−^) to hydrogen peroxide (H_2_O_2_). Hydrogen peroxide can then be converted to water by peroxiredoxin (PRDX), catalase (CAT), glutathione peroxidase (GPX), or thioredoxin (TXN). Small non-enzymatic antioxidants such as vitamin C, vitamin E, uric acid, bilirubin, β-carotene, and plant polyphenols are also important in free radical scavenging [14]. Hydrogen peroxide generated by mitochondria can be distributed to other organelles, including the endoplasmic reticulum (ER) via aquaporin 11. Hydrogen peroxide can act as a signaling agent at physiological levels, activating numerous pathways including NF-κB p65, nuclear factor erythroid 2-like 2 (NFE2L2)/KEAP and c-Src/EGFR/ERK/Akt [20]. 

Other intrinsic sources of ROS include non-phagocytic NADPH oxidase 4 (NOX-4), the peroxisome or endoplasmic reticulum (ER). Hydrogen peroxides can be converted to highly damaging hydroxyl radicals (•OH) in the presence of iron by Fenton reactions [16,21]. ROS can damage DNA, cell membranes, and lipids and also shorten telomeres, causing the DNA damage response (DDR), lipid peroxidation, or cellular aging. Both telomeric and telomere-independent DNA damage result in the activation of ATM (ataxia–telangiectasia-mutated) kinase (by DNA double-strand breaks) and ATR (ATM- and Rad3-related) kinase (by DNA single-strand breaks). These DDR kinases phosphorylate and stabilize p53, resulting in increased expression of its transcriptional target p21^WAF/CIP1^ (cyclin-dependent kinase inhibitor 1A (CDKN1A)). Single-strand breaks in DNA due to ROS are thought to stimulate p38MAPK signaling and activation of the p53-independent p16^INK4a^-Rb (CDKN2A) pathway, which inhibits the CDK4 and CDK6 phosphorylation of retinoblastoma protein (Rb) and the release of the transcription factor E2F [15]. While p21^WAF/CIP1^ initiates cell cycle arrest in the G1/S phase or G2/M phase DNA damage checkpoint and cellular senescence, p16^INK4a^-Rb stabilizes a G1/S cell cycle arrest, making senescence irreversible [22]. Proliferative arrest due to the DDR is thought to protect against oncogenesis, as well as maintain genomic integrity by enabling successful DNA repair prior to the cell re-entering the cell cycle. Non-prolonged cellular stress can result in cellular quiescence, a (reversible) arrest of proliferation in the G0 phase, whereas severe stress can trigger apoptosis [15]. 

Ataxia–telangiectasia-mutated kinase is also found in the mitochondria, endosomes, and peroxisomes and has non-canonical roles in autophagy regulation during hypoxia, oxidative stress, or glucose deprivation [23]. Activation of ATM inhibits vacuolar ATPase (vATPase), causing an increase in lysosomal pH. Because acidification is required for lysosomal lipase, the activity of the autolysosome is impaired, contributing to the expansion of lysosome numbers, retention of lipofuscin, and increased β-galactosidase in senescence. Assembly of vATPase is inhibited by mTORC1 under nutrient-rich conditions, which can also contribute to the inhibition of autophagic flux [24,25,26,27]. Inhibition of autophagic flux and mitophagy can contribute to mitochondrial dysfunction, mtROS generation, mitochondrial DNA (mtDNA) oxidation, and accumulation of damaged mitochondria [15]. Mitochondrial DNA is more prone to damage from oxidative stress than nuclear DNA, as mtDNA is not protected by introns or histones and is located near the electron transport chain where mtROS are generated [28]. 

Release of oxidized mtDNA from damaged mitochondria or telomeric nuclear DNA into the cytoplasm activates the cytosolic DNA-sensing cyclic GMP-AMP synthase stimulator of interferon genes (cGAS-STING) pathway [29,30,31]. This has the downstream effect of stimulating interferon regulatory factor 3 (IRF3) and TLR/nuclear factor κB (NF-κB) inflammasome signaling, which can promote pro-survival pathways in cellular senescence. These include the release of exosomes carrying programmed death ligand 1 (PD-L1) and mtDNA, adipocyte and pancreatic β-cell senescence and functional decline, and inhibition of beige/BAT mitochondrial thermogenesis [29,30,31]. cGAS-STING release is a feature of cellular metabolic stress induced by palmitate or hydrogen peroxide exposure in both aged mice and aged HFD obese mice. SCAPs not only affect post-mitotic terminally differentiated cells such as mature adipocytes but also their pluripotent progenitors and immune cells involved in programmed death pathways. Inhibition of apoptosis, accumulation of senescent cells, and the inability to remove and replace them with healthy new cells contribute to hypertrophic obesity, T2DM, and premature aging [32,33,34,35]. 

### 4.1. Epigenetic Control of DNA Expression and Obesity

Generation of ROS in obesity and aging can also influence the *epigenetic* control of DNA expression by causing aberrant nuclear and mitochondrial DNA methylation and histone acetylation [36,37,38]. Differentially methylated regions (DMRs) can result from the DNA oxidation products 5-methylcytosine (5mC), hydroxymethylcytosine (5hmC), or 8-oxo-2′-deoxyguanosine. In epigenome-wide association studies (EWAS), DNA methylation was estimated to contribute to 10% of the variance of the BMI in adults, with methylation of *ABCG1* and *CPT1A* CpG sites having the most powerful contributions. The epigenetic control of DNA also involves non-coding RNA (ncRNA), including microRNA (miRNA), circular RNA (circRNA), and long non-coding RNA (lncRNA), which inhibit the translation of genes by mRNA. 

The role of the epigenome in the modern obesity epidemic is important, as despite hundreds of obesity-susceptibility loci being found in genome-wide association studies (GWAS) of common or polygenic obesity, carriage of these germline gene variants only accounts for 6% of the BMI variance in the adult population [36,37,38]. These include genes for the neurohormonal regulation of appetite and satiety (*BDNF*, *MC4R*, *NEGR*), energy substrate and lipid metabolism (*FTO*, *RPTOR*, *MAP2K5*), insulin secretion and activity (*TCF7L2*, *IRS1*), and adipogenesis (*CEBPA*, *PPARG*, *BMP2*, *HOXC*/*miR196*). The small phenotypical effect is related to the low penetrance of single nucleotide polymorphisms in obesity genes and the epigenetic influence of modifiable lifestyle risk factors on CpG island DMRs, including food, social, and built environments. For example, the fat mass and obesity-associated (FTO) risk allele is the most common obesity gene in European and Asian populations, accounting for an increase in BMI of 0.35 kg/m^2^ per allele. However, this effect could be attenuated by exercise or exacerbated by the consumption of unhealthy fried foods [36,37,38,39].

Some isolated, founder or discovery human populations with increased carriage of homozygous and heterozygous gene mutations have a higher susceptibility to obesity [36,37,38]. For example, the loss-of-function adenylate cyclase 3 (*ADCY3*) gene variant in Greenlanders is associated with lipogenesis, obesity, and T2DM due to impaired cAMP signaling in adipocytes. The cyclic-AMP-responsive element-binding protein 3 regulatory factor (*CREBRF*) gene variant is common (minor allele frequency = 0.26) in Polynesian Samoans (BMI, 1.36–1.45 kg/m^2^ per allele), which may provide a survival advantage in conditions of nutrient deprivation [36,37,38]. However, recent clinical studies suggest carriage of the CREBRF^R457Q^ variant is significantly associated with increased lean body mass (muscle/bone) and vertical height in adult Samoans, lower myostatin levels in males, and *decreased* risk of T2DM (odds ratio = 0.59) or gestational DM, further complicating the *thrifty gene hypothesis* of obesity. A genetically modified pig model with CREBRF^R457Q^ variant fed a commercial diet ad libitum showed increased fat deposition via SAT preadipocyte differentiation and adipocyte hyperplasia and higher SAT antioxidant levels of CAT, GPX, and SOD, with decreased markers of SAT ROS generation and oxidative stress (malondialdehyde (MDA), 4-hydroxy-nonenal (HNE)). This was associated with lower fasting serum glucose and triglycerides but higher fasting serum insulin levels. In contrast, wild-type CREBRF pigs developed an expansion of VAT volume with associated adipocyte hypertrophy, impaired glucose tolerance, insulin resistance, oxidative stress, and lipid peroxidation. The CREBRF^R457Q^ variants developed metabolic changes that resembled the human CREBRF^R457Q^ metabolic phenotype as compared to orthologous murine models, which showed limited metabolic effects. One of the functions of CREBRF is to inhibit the endoplasmic reticulum (ER) unfolded protein response (UPR). It does so by sequestering the ER membrane-bound transcription factor CREB3 and preventing its nuclear translocation and activation of UPR element-containing promoters in response to ER-Golgi stress. CREB3 has a similar structure to activating transcription factor 6 (ATF6), which senses ER proteotoxic stress, and the ER-bound sterol regulatory element-binding protein (SREBP), which regulates fatty acid and cholesterol metabolism [40,41,42,43]. This is discussed further in Section 4.6.

### 4.2. Diet, Postprandial Oxidative Stress, and Loss of Metabolic Flexibility

**Figure 1:**
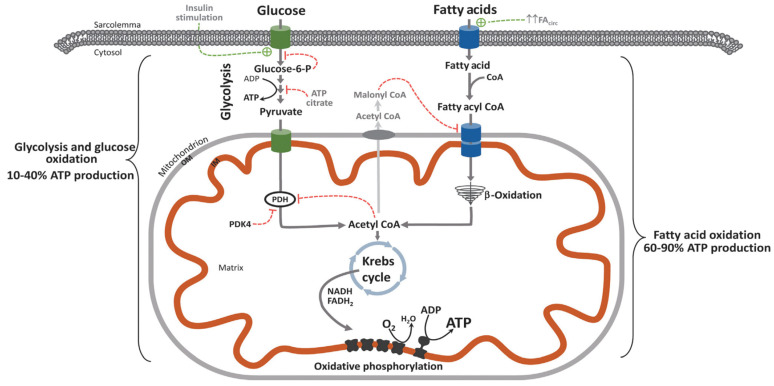
The Randle and reverse Randle effects in the cyclical inhibition of substrate metabolism during feeding (glucose oxidation) and fasting/exercise (β-oxidation of FFAs), which is dysregulated by insulin resistance, hyperglycemia, and hypernefemia. Glucose is transported (stimulated by insulin) and partially metabolized through glycolysis prior to conversion of pyruvate in the mitochondrion to acetyl-CoA by pyruvate dehydrogenase (PDH). Acetyl-CoA is oxidized through the Krebs cycle to generate reducing equivalents (NADH and FADH_2_). Uptake of fatty acids is primarily dictated by plasma concentrations, and fatty acid metabolism through β-oxidation in the mitochondrial matrix contributes the largest contribution to ATP production under normal conditions. NADH and FADH_2_ generated from substrate oxidation are used in the process of oxidative phosphorylation through the coupled reactions of the electron transport chain and an ATP synthase. Several points of regulation are depicted; red dashed lines indicate points of negative feedback (inhibition), and green dashed lines indicate points of positive feedback (see text). FA, fatty acids; OM, outer membrane (mitochondrial); IM, inner membrane. Reproduced from Berthiaume 2016 [48].

Modern urbanized humans spend a large proportion of the day in post-prandial oxidative stress due to high saturated fat and Western-style inflammatory diets [44]. Post-prandial oxidative stress is more pronounced in obesity/metabolic syndrome and exacerbated by the depletion of enzymatic antioxidant defense systems (GPX-1, GPX-3, CAT, PRDX, TXN) and small non-enzymatic antioxidants [44]. Such macronutrient excess and elevated circulating levels of non-esterified saturated fatty acids (SFA) or *hypernefemia* contribute to skeletal muscle mitochondrial stress, incomplete FAO oxidation, and peroxide formation, leading to acylcarnitine build-up and insulin resistance. Beta-oxidation of long-chain fatty acids by mitochondria is less efficient than pyruvate oxidation. Consequently, there is an increase in mitochondrial [acetyl-CoA]/[CoA] and [NADH]/[NAD^+^], blockade of glycolysis at the level of pyruvate dehydrogenase (PDH), and, to a lesser extent, phosphofructokinase (PFK) and hexokinase II (*Randle effect*) [45]. Conversely, after a meal, glucose oxidation normally results in the formation of malonyl-CoA via the Krebs cycle, which blocks carnitine palmitoyltransferase (CPT1) activity, preventing FAO (*reverse Randle effect*). Hyperinsulinemia, hyperglycemia, and hypernefemia are features of impaired substrate utilization and metabolic inflexibility when switching between fasting and fed states (*Randle cycle*). This results in the diversion of up to 30% of glucose from glycolysis and pyruvate production into the cytosolic polyol pathway. Activation of the polyol pathway is associated with protein glycation, formation of advanced glycation end-products (AGEs), 4-hydroxy-nonenal glutathione (HNE-GSH) adducts, and loss of GPX activity. A feed-forward effect is created of further fructose/sorbitol synthesis; NADH/NAD^+^ redox imbalance; AGE-RAGE interaction, oxidative, cellular, and mitochondrial stress; and activation of (NF-κB) inflammasome pathways (discussed further in Section 8) [45,46,47,48] (Figure 1).

### 4.3. Oxidative Stress and Cellular Senescence

Oxidative stress is a feature of both obesity and premature aging. Elevated levels of the DNA oxidation marker 8-hydroxy-2-deoxyguanosine in hypertrophic adipocytes actually *predate* the development of adipose tissue inflammation, insulin resistance, and glucose intolerance [15]. Although telomeres make up only 0.025% of the overall genome, oxidation of telomeric guanine bases is sufficient to produce rapid p53-induced cellular senescence within 30 minutes of damage [49]. The persistent generation of ROS has a feed-forward effect of ongoing DNA damage, DDR, and stress-induced cellular senescence with progressive adipocyte hypertrophy [16]. The human progeroid syndrome of pathological aging and obesity-related stress-induced pancreatic β-cell senescence both share a similar phenotype to DNA excision repair gene *Ercc1* deficient mice, which is related to prolonged DNA damage and the activation of DDR pathways [33]. 

### 4.4. Pathways to Cellular Senescence and SASP

There are numerous interrelated contributors to mitochondrial dysfunction and stress-induced cellular senescence in obesity [49,50,51,52,53,54,55,56,57,58]. These include the following:

1. Excessive macronutrient supply with inadequate micro/phytonutrient intake;
2. Activation of oncogenic signaling pathways (Ras/Raf/MAPK, JAK/STAT, PI3K/AKT/mTOR, NF-κB);
3. Adipocyte hypoxia and ischemia;
4. Accumulation of advanced glycation end-products (AGE) or ceramide;
5. Decreased NAD^+^/NADH and GSH/GSSG ratios;
6. ER stress and UPR;
7. Epigenetic modifications (acetylation of histones and nuclear transcription factors);
8. Paracrine secretion of cytokines;
9. Defective chaperone-mediated, selective-, or macro-autophagy and intracellular retention of lipids, damaged organelles, unfolded proteins or GATA4/p62;
10. Accumulated telomere-associated DDR foci (TAFs), which are length-independent DNA damage sites within telomeres associated with ATM and other PI3K protein kinase-related phosphorylation of histone 2 AX (γH2AX) and p53-binding protein 1 (53BP1) [49,50,51,52,53,54,55,56,57,58].

Continued exposure to these intrinsic or extrinsic risk factors leads to suppression of human antigen R (HuR) RNA-binding protein, NAD^+^-dependent protein deacetylase silent information regulator two homolog 1 (sirtuin 1, SIRT-1), and heat shock factor 1 (HSF-1), as well as activation of hypertrophic adipocyte senescence and a SASP [49,50,51,52,53,54,55,56,57,58]. SIRT-1 is required for the expression and transcriptional activity of HSF-1. HSF-1 is a transcription factor that, when released, translocates to the nucleus and activates the nuclear heat shock element, increasing the transcription of heat shock proteins (HSP27, HSP60, HSP70, and HSP90). Intracellular HSPs are chaperones required for refolding of unfolded or misfolded proteins and resolution of the UPR during ER stress [13]. HSF-1 suppresses c-Jun NH2-terminal kinase (JNK) and NF-κB inflammatory pathways and inhibits transcription of the tumor necrosis factor alpha (TNF-α) gene [59]. Loss of SIRT-1 is thus intimately related to the failure of resolution of the UPR and ER stress and activation of NF-κB. 

### 4.5. SIRT-1 and Cellular Senescence

NAD^+^ is required for SIRT-1 activity and also DNA repair by poly-ADP ribose polymerases (PARPs) [50]. DNA repair by PARPs consumes NAD^+^, and thus, ongoing adipocyte DNA damage indirectly inhibits SIRT-1 [50]. Genotoxic stress and the DDR increase both p53 and cell cycle and apoptosis regulator protein 2 (CCAR2), which, respectively, inhibit SIRT-1 transcription and SIRT-1 activity. SIRT-1 protein expression is also downregulated by several miRNAs, including miR-9, miR-34a, miR-133, miR-143, miR-132, miR-146, miR-155-5p, miR-181a, and miR-199a-5p [60,61,62]. 

SIRT-1 has the epigenetic effect of deacetylating histones and non-histone proteins (including transcription factors and non-transcriptional regulatory proteins) [60,61]. SIRT-1-mediated deacetylation results in alterations of gene expression, chromatin compaction, and inhibition of the following:

- Inflammation (NF-κB p65 deacetylation and reduced miR-155);
- Oxidative stress (NFE2L2 deacetylation, FOXO3 deacetylation, increased SOD2, CAT, GSH);
- UPR-mediated inflammation (PERK, CHOP, caspase-12, spliced XBP1, SMAD3, ATF4);
- Adipocyte cellular senescence (p53/p21^CIP1^ deacetylation);
- Hypoxia signaling and glycolysis (HIF-1α deacetylation);
- Adipocyte differentiation, lipid synthesis, VAT fat deposition (PPARγ deacetylation, SREBP);
- Macrophage COX2 expression and cytokine-induced inflammation (deacetylation of P300-mediated activator protein 1 (AP-1) acetylation) [60];
- MCP-1/CCL-2 and macrophage chemotaxis (deacetylation of H3K9) [63];
- TNF-α (histone H3K16 deacetylation) and TNF-α and interleukin 6 (IL-6) expression (H3K9 or high-mobility group box 1(HMGB1) deacetylation);
- Transgenerational inheritance of high-fat diet (HFD)-induced obesity [60,64].

SIRT-1 mediated deacetylation activates the following:

- AMP-activated protein kinase (AMPK) (deacetylation of liver kinase B1 (LKB1));
- Mitochondrial respiration and biogenesis (deacetylation of CCAAT/enhancer-binding protein alpha (C/EBPα));
- Fatty acid β-oxidation (FAO) and mitochondrial biogenesis (deacetylation of PGC-1α);
- Reverse cholesterol transport into high-density lipoproteins (deacetylation of liver X receptor (LXR));
- Hepatic bile acid production (deacetylation of farnesoid X receptor (FXR));
- Adipocyte triglyceride lipolysis by ATGL (deacetylation of FOXO1);
- Autophagy (deacetylation of FOXO1, ATG5/7/8 during glucose starvation);
- Autophagy (deacetylation of microtubule-associated protein 1 light-chain 3 beta (MAP1LC3B/LC3B));
- Heat shock response (deacetylation of HSF-1);
- Anti-inflammatory IL-10 secretion (deacetylation of histone H3K9 and H4K16);
- Adiponectin gene (*ADIPOQ*) transcription (deacetylation of FOXO1);
- Hepatic triiodothyronine (T3) activity and FAO (deacetylation of thyroid hormone receptor β1 (TRβ1));
- Browning of WAT (deacetylation of PPARγ, activation of AMPK, FGF21);
- Adipocyte-derived IL-4 production (deacetylation of nuclear factor of activated T cells 1 (NFATC1)) [60,63,65,66,67,68,69].

SIRT-1 and p53 exist in a reciprocal negative feedback loop, with p53 binding to the P53 response element in the *SIRT1* promoter, thereby inhibiting *SIRT1* transcription. Acetylation of p53 leads to increased expression of p21^CIP1^, which is inhibited by p53 deacetylation by SIRT-1 protein. Loss of SIRT-1 transcription or post-translational modification of its activity is central to the development of p53-induced cellular senescence and metaflammation during obesity [60].

### 4.6. ER Stress, UPR, Apoptosis, and Senescence

**Figure 2:**
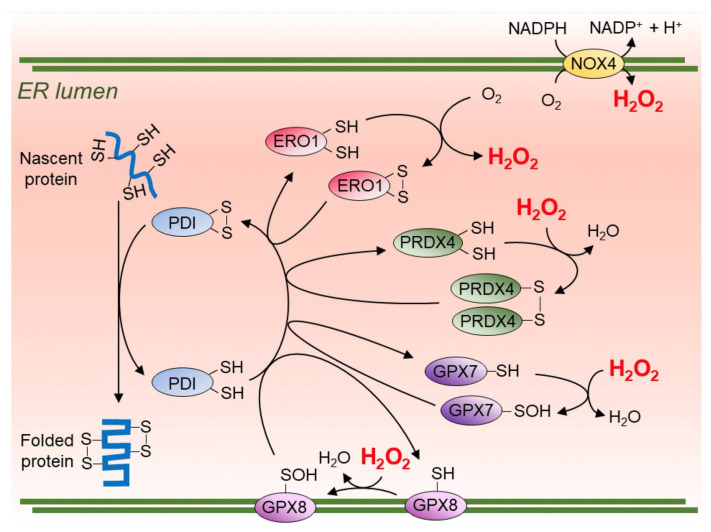
Schema of pathways producing and utilizing endoplasmic reticulum (ER)-derived H_2_O_2_. Reduced thiols (-SH) of cysteine residues in nascent proteins are oxidized by catalytically competent protein disulfide isomerase (PDI) to promote protein folding with native disulfide bonds (S-S). PDI can be re-oxidized by ERO1, with flavin adenine dinucleotide (FAD) embedded in its active site, which reduces molecular oxygen (O_2_) to hydrogen peroxide (H_2_O_2_). ER-resident antioxidants PRDX4, GPX7, and GPX8 oxidize their catalytic domain by utilizing H2O2 (-SOH = sulfenic acid) and mediate “the disulphide relay” to PDI. NOX4 produces H_2_O_2_ over superoxide on the ER membrane, unlike other NOX family proteins. Reproduced from Konno et al. (2021) [70].

Sublethal ER stress and an adaptive UPR can be useful for ER and mitochondrial hormesis, matching physiological demands for protein and hormone production and promoting cell survival [70]. One-third of the entire proteome enters the ER from ribosomes for processing, folding, glycosylation, assembly of multimers, or packaging into vesicles for secretion. Oxidative folding of proteins in ER is dependent on hydrogen peroxide generated from molecular oxygen by membrane-localized NOX4, ascorbate oxidase, or ER flavoprotein oxidoreductin 1 (ERO1). These are linked to resident antioxidant enzymes (PRDX4, GPX7, GPX8) and the protein disulfide isomerases (PDI), which fold proteins by creating linked disulfide bonds in unfolded nascent proteins. The ER is devoid of DNA and thus is able to maintain a highly oxidative environment, with the ER producing or consuming up to 45% of the hydrogen peroxide in the body. In contrast, the nucleus, cytoplasm and mitochondria maintain a strong reductive environment which helps to protect the genome from oxidation [70] (Figure 2).

Up to 25% of the oxygen use in the cell is by ERO1 [70,71]. However, during ER stress, this carefully coordinated redox system is disrupted, causing accumulation of unfolded proteins with distention of the ER lumen, increased ROS production, and depletion of intracellular glutathione (GSH) by oxidation. This results in the dissociation of the luminal ER HSP70 chaperone-binding immunoglobulin protein (BiP), which unlocks the three transmembrane ER stress-sensing proteins, inositol-requiring enzyme 1 (IRE1α), protein kinase RNA-like ER kinase (PERK), and activating transcription factor 6α (ATF6α). These orchestrate an *adaptive* UPR to reduce ER stress and maintain cellular proteostasis [70,71]. 

**Figure 3:**
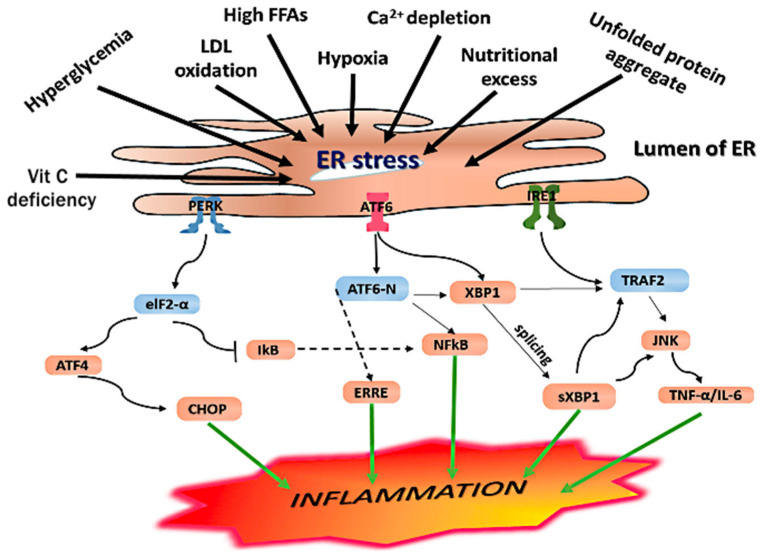
Maladaptive UPR and endoplasmic reticulum (ER) stress-mediated inflammation. Under ER stress, the UPR is activated, and this leads to activation of the three principal UPR signaling transmembrane receptor proteins, including IRE-1, PERK, and ATF6. Activation of the IRE-1 leads to the splicing of the mRNA of a transcription factor XBP1 and subsequent expression of sXPB1, a highly active transcription factor for the release of ER-resident enzymes and molecular chaperones. As a result, it leads to activation of NF-κB and CHOP, resulting in increased expression of proinflammatory gene products. Likewise, activated IRE-1 recruits TRAF2, and this complex causes activation of downstream signaling of kinases, including JNK and NF-κB, which induce production of inflammatory cytokines and trigger inflammation. These inflammatory kinases then phosphorylate and activate downstream mediators of inflammation. Phosphorylation of PERK/eIF2α downstream signaling pathway results in uncoupling of NF-κB from IkB. As a result, NF-κB translocates into the nucleus, where it activates expression of genes encoding proinflammatory cytokines, including IL-1, IL-6, and TNF-a, resulting in persistent inflammatory response. On the other hand, autophosphorylation of PERK initiates activation of eukaryotic initiation factor 2 (eIF2α), which further undergoes phosphorylation, resulting in translational attenuation of protein synthesis. Similarly, this downstream phosphorylation of eIF2α leads to increased expression of ATF4 and translocation into the nucleus, where it binds to the UPRE, resulting in transcriptional modification of CHOP, a proapoptotic gene transcription factor that initiates inflammation as well as apoptosis. IRE-1 recruits TRAF2 and causes activation of downstream signaling of kinases, including JNK and NF-κB, inducing the production of inflammatory cytokines. PERK phosphorylates eIF2α, which leads to the activation of NF-κB and CHOP to further promote the expression of the inflammatory gene. ER stress leads to dissociation of TRAF from TRAF2-procaspase 12 complex, which is located on the ER membrane, leading to activation of caspase 12. At the same time, IRE1–JNK complex recruits TRAF2 leading to the formation of the IRE1–JNK–TRAF2 complex. The ATF6 pathway also activates NF-κB, further intensifying the expression of inflammatory genes, which secrete more cytokines. Reproduced from Amen et al. (2019) [14].

IRE1α induces transcription of foldases, chaperones, and components of ER-associated protein degradation (ERAD), as well as expansion of ER luminal capacity [72,73]. The endoribonuclease activity of IRE-1α digests messenger RNAs (mRNA) associated with apoptosis and lipogenesis by regulated IRE1-dependent decay (RIDD). PERK inhibits ER protein translation via eukaryotic initiation factor 2 (eIF2α) phosphorylation and activates ATF4, ATF3, and C/EBP homologous protein (CHOP/growth arrest- and DNA damage-inducible gene 153 (GADD153)), as well as the transcription of genes, which regulate apoptosis (death receptor 5 (DR5)), autophagy (ATG5, ATG12, ATG8), the antioxidant response (NFE2L2), and amino acid metabolism. PERK activates ATF5, which increases the transcription of thioredoxin-interacting protein (TXNIP). TXNIP negatively regulates thioredoxin (TRX) and activates the NLRP3 inflammasome. The PERK-eIF2 axis also activates NF-κB signaling by suppressing the inhibitor of κBα (IκB), a process that is separate from the direct activation of NF-κB by ATF6. ATF6α is also trafficked from the ER to the Golgi and undergoes productive cleavage by sequential Golgi site 1 and site 2 serine proteases (S1P, S2P). The resulting cytoplasmic bZIP domain of ATF6α then translocates to the nucleus, acting as a transcription factor to enhance the expression of X-box-binding protein 1 (XBP1), ER folding chaperones (BiP/GRP78, GRP94, PDI), and ERAD-associated proteins (ubiquitin ligases). This promotes refolding or ERAD of misfolded/unfolded proteins and can decrease ER stress by clearing these accumulated proteins from the ER lumen. ERAD involves the removal of ubiquitinated proteins by (I) ubiquitin–proteasome-dependent ERAD or (II) the formation of an autophagosome and export to lysosomes via autophagy [72]. ERAD proteins also control calcium homeostasis, sterol biosynthesis, and interactions between ER and other organelles, including mitochondria. Apart from luminal accumulation of unfolded protein aggregates, ER stress can also be exacerbated by hypoxia, excessive ROS, mitochondrial dysfunction, inflammatory cytokines, impaired processing of glycoproteins by the lectin-type chaperones calnexin/calreticulin, saturation of ER membranes with cholesterol, TLR ligands, chronic saturated NEFA and hyperglycemia exposure and impaired antioxidant responses, or autophagy of proteins. ER stress due to excessive amino acids, palmitate or fructose can increase lipogenesis by mTORC1-SREBP activation, and impair lipophagy by mTORC1-ATF4-CHOP4 activation and inhibition of lysosomal-associated membrane protein 2 (LAMP2) [14,74] (Figure 3).

Inhibition of protein translation cannot be maintained indefinitely during ER stress, and if the adaptive chaperone and heat shock responses are impaired, then proteins such as proinsulin, procollagen, and adiponectin are not properly folded or re-folded in the ER lumen [75]. Ongoing ER stress and unresolved luminal unfolded protein build-up leads to *maladaptive* UPR and inflammasome activation [14]. Activation of NF-κB signaling increases the release of inhibitors of apoptosis proteins (IAP), including c-IAP1, c-IAP2, and XIAP, and members of the Bcl family Bcl-2/Bcl-xL, which are pro-survival elements involved in cellular senescence. The IAP family inhibits the executioner cysteine proteases caspase-3 and caspase-7, and Bcl-2/Bcl-xL inhibits the insertion of the pore-forming agents BAK/BAX into the mitochondrial outer membrane, preventing mitochondrial-mediated apoptosis. Severe ER stress can result in cellular apoptosis mediated by CHOP via activation of BAK, BAX increased MOMP, and decreased expression of the anti-apoptosis protein Bcl-2 [14]. Prolonged IRE1α stimulation can also mediate apoptosis and autophagy by activating TRAF2/ASK1/JNK/P38 MAPK pathways [72,73]. Recently, it has been shown that sublethal mitochondrial apoptosis signaling promotes cellular senescence and SASP. This involved an increase in MOMP and release of cytochrome c and mtDNA in a small proportion of mitochondria and crosstalk between the ER and mitochondria [76]. Furthermore, hyperglycemia creates a sublethal level of ER stress, which increases insulin secretion via IRE-1α and pancreatic β-cell proliferation via ATF6, while sustained stimulation of all three UPR pathways by saturated FFAs culminates in pancreatic β-cell apoptosis [77]. ER glucolipotoxicity, due to hyperglycemia combined with saturated FFAs (palmitate), has a synergistic effect on β-cell apoptosis. This is because opposing UPR signals, such as CHOP and RIDD, converge on DR5/tumor necrosis factor-related apoptosis-inducing ligand receptor 2 (TRAILR2), which determines the cell fate based on the type, intensity, and duration of ER stress [72] (Figure 3).

HFD-induced obesity in C57BL/6 mice resulted in ER stress, intracellular free cholesterol accumulation, lipogenesis, NF-κB signaling, and a proinflammatory secretion profile of adipokines (resistin, TNF-α) in hypertrophic adipocytes, as well as systemic dyslipidemia and hyperglycemia [78,79]. Inhibition of GRP78 and CHOP by the small-molecule ER chemical chaperones tauro-ursodeoxycholic acid (TUDCA) or 4-phenyl butyric acid (4-PBA) alleviated ER stress and improved adipose tissue inflammation and the above metabolic derangements. 4-PBA is a derivative of the gut microbial fermentation-derived short-chain fatty acid butyrate and protects unfolded proteins from aggregating in the ER lumen, promoting their refolding. TUDCA is a bile acid taurine conjugate derived from ursodeoxycholic acid, which inhibits the oxidized LDL activation of CHOP in the ER. Both humans and C57BL/6 mice develop ER stress, cellular senescence, accelerated aging, functional and cognitive decline, hypertrophic obesity, and obesity-related comorbidity associated with Western or cafeteria-type diets and sedentary lifestyles. This is why C57BL/6 mice are a particularly useful model for human obesity and aging research. However, female C57BL/6 mice are more resistant to diet-induced obesity and development of metabolic derangement as compared to male C57BL/6 mice [78,79].

### 4.7. Transmissible Senescence, UPR, and SASP

**Figure 4:**
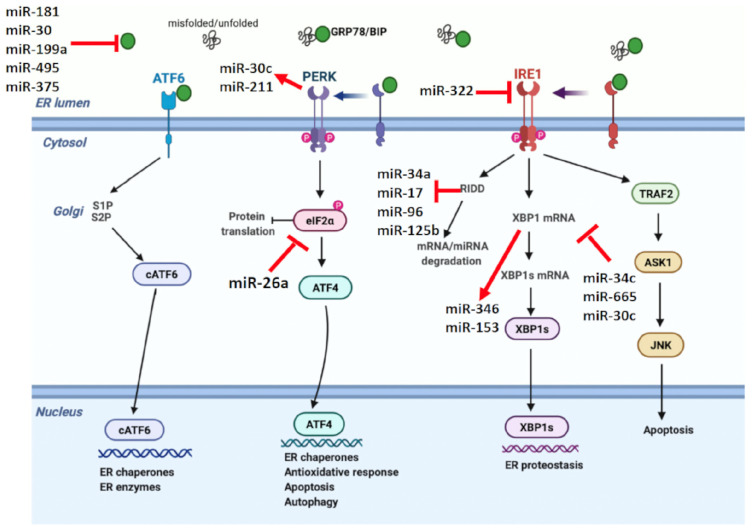
Unfolded protein response signaling with their regulating microRNAs. miRNAs have a crucial role in shaping the UPR, while miRNA expression is also regulated by UPR. miR-181, miR-30, miR-199a, miR-495, and miR-375 negatively regulate GRP78. On the other hand, miR-322 suppresses IRE1. XBP1 and RIDD signaling, members of IRE1 branch in UPR, regulate miR-153, miR-346, miR-34a, miR-17, miR-96, and miR-125b. Additionally, miR-34c, miR-665, and miR-30c are known to target XBP1, while miR-26a suppresses eIF2α, reducing the protein translation. Certain miRNAs also form a link between UPR branches. In this direction, PERK-mediated miR-30c activation inhibits XBP1 pathway and establishes a negative crosstalk between PERK and IRE1 branches of UPR. Reproduced from Demirel-Yalciner (2022) [80].

During obesity, senescent cells develop an inflammatory UPR in the ER directed to continuous activation of the NLRP3 inflammasome [12]. This represents a *transmissible* ER stress/UPR/SASP auto-amplification loop [52,56], related in part to NLRP3-associated caspase-1 cleavage of SIRT-1 and suppression of the SIRT-1/HSF-1/HSP molecular axis [12]. Transmissible ER stress can also be promoted by circulating miRNAs, which target specific components of the adaptive UPR. For example, miR-181a can promote peroxide-induced senescence by inhibiting the protective effects of ER PDI. During obesity, increased miR-181a and miR-199a suppress ER HSP70 chaperone BiP/GRP78 expression; in NAFLD, miR-30c downregulates XBP-1 expression, and miR-26a reduces eIF2α. MicroRNAs themselves may be regulated by ER stress UPR due to cleavage by IRE-1α RNase and degradation by RIDD [61,80,81] (Figure 4).

Although senescent cells do not proliferate, they are metabolically active with a vigorous SASP, including active secretion of the following:

- Pro-inflammatory cytokines (IL-1α, IL-1β, IL-6, IL-8, TNF-α, activin A, osteopontin);
- Chemokines (CXCL-1/3, CXCL-8/IL-8, CXCL-10, MCP-1/CCL2, MIP-1α/CCL3, RANTES/CCL5);
- Proteases and cell surface molecules (plasminogen inhibitors/activators (PAI-1/2, tPA, uPA), matrix metalloprotease enzymes (MMP-1, -3, -10, -12), and intercellular adhesion molecule 1 (ICAM-1);
- Growth factors (VEGF, TGF-β, PDGF, GM-CSF, HGF);
- Bioactive lipids (oxidized lipid mediators, prostanoids, bradykinins, ceramides);
- Extracellular vesicles (EVs);
- Damage-associated molecular patterns (DAMPs) (HMGB1, serum amyloid A1 (SAA1));
- Nucleotides (mitochondrial DNA, miRNA, nuclear DNA) [50,52,55,56,57].

Increased lysosomal numbers and lysosomal-derived senescence-associated β-galactosidase (SA-β-gal) are features of cellular senescence [27,29,30,31]. These are associated with impairment of lysosomal function, autophagy, and mitophagy and poor substrate degradation, including lipids (which generate lipofuscin), proteins, and damaged organelles. Cellular senescence markers include phospho-p53, p21^CIP1^, p16^INK4a^, SA-β-gal, the phosphorylated form of histone H2AX (γ-H2AX), intracellular lipofuscin bodies or cellular, and nuclear hypertrophy [15,82]. Accumulation of these senescence markers and metaflammation in both subcutaneous adipose tissue (SAT) and VAT is a feature of obesity in animal models and humans. Senescence in adipocyte precursors impairs the hyperplastic expansion of SAT storage, which promotes adipocyte hypertrophy, lipotoxicity, and insulin resistance [15,82]. The degree of hepatocyte senescence correlated with the severity of NAFLD and was characterized by impaired mitochondrial FAO and accumulation of intracellular lipids in obese rats. Liver biopsies from patients with NAFLD displayed features of senescence with shorter hepatocyte telomere length, elevated p21^CIP1^, and enlarged nuclei [15,82]. Clearance of p16^INK4a^ senescent cells in the pancreas improved glucose metabolism and insulin secretion, and clearance of p21^CIP1^ cells from VAT was shown to improve adipogenesis and insulin sensitivity and decrease VAT volume and macrophage infiltration in murine obesity models [15,82]. 

**Figure 5:**
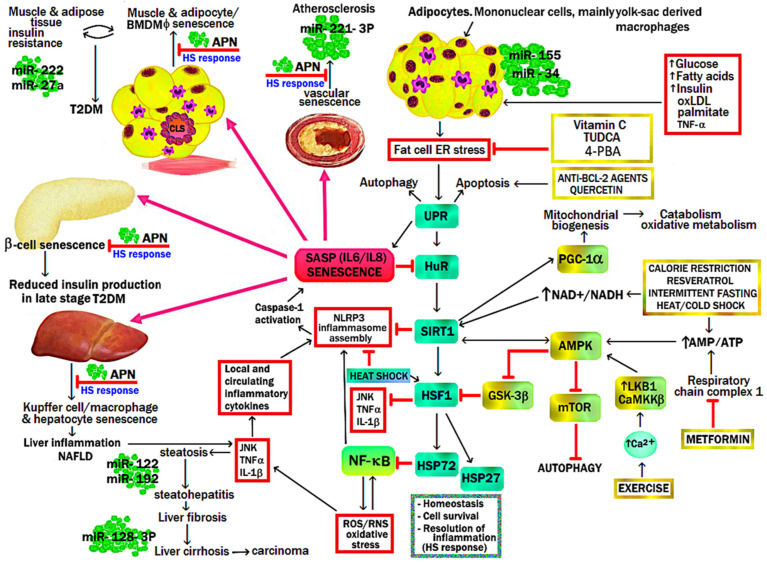
Unifying hypothesis for transmissible ER stress, UPR, adipocyte SASP, NLRP3 inflammasome activation and inhibition of human antigen R (HuR) RNA-binding protein, SIRT-1, heat shock factor 1 (HSF-1), adiponectin (APN), and impaired heat shock (HS) response during chronic metabolic stress in obesity. Under conditions of nutrient excess, insulin signaling, hypoxia, and ER stress, hypertrophic adipocytes fail to undergo apoptosis and thus develop a senescence-associated secretory phenotype (SASP). This is associated with loss of HuR-SIRT1-HSF1 pathways, impaired HS response, prolonged activation of the NLRP3 inflammasome, and release of detrimental exosomal miRNA. The WAT SASP and ER stress can spread to other tissues, including pre-adipocytes, WAT-infiltrating bone marrow-derived macrophages (BMDMΦ), skeletal muscle, vascular endothelium, pancreatic β cells, and Kupffer cells/hepatocytes. This leads to accelerated obesity, atherosclerosis, insulin resistance, T2DM, and NASH/liver cirrhosis. A key feature of SASP in hypertrophic adipocytes is the recruitment of BMD monocytes and their polarization to metabolically activated macrophages via cytokine, chemokine, and exosomal microRNA (miR-34a, miR-155) release and formation of crown-like structures (CLS) in WAT. Lean WAT produces exosomal adiponectin (APN), which, in the presence of a functional heat shock response, inhibits vascular, pancreatic β-cell, hepatocyte, and WAT metaflammation and senescence. In obese VAT, exosomal adiponectin is decreased by 40-fold. Impaired multimerization of adiponectin due to vitamin C deficiency contributes to ER stress and UPR in hypertrophic obesity and loss of HMW adiponectin secretion. Hypoxia in hypertrophic obesity also impairs oxygen and ascorbate-dependent ER protein folding, leading to the UPR response and further ER stress. During adipocyte hypertrophy, ER stress can be improved by vitamin C, tauro-ursodeoxycholic acid (TUDCA), or 4-phenylbutyric acid (4-PBA) administration [22,23,24,25,26,27,28,29,30,31,32,33,34,35,36,37,38,39,40,41,42,43,44,45,46,47,48,49,50,51,52,53,54,55,56,57,58,59,60,61,62,63,64,65,66,67,68,69,70,71,72,73,74,75,76,77,78,79,80,81,82,83,84,85,86,87,88,89].

Lean WAT is particularly important for plasma and exosomal adiponectin secretion, with an intact heat shock response required for adiponectin receptor activity in target tissues [83,84]. During adipocyte hypertrophy, downregulation of adiponectin mRNA and protein expression occurs. There is also impairment of the folding, assembly, and secretion of high-molecular-weight (HMW) adiponectin multimers by lysyl- and prolyl-hydroxylases and disulfide bond A oxidoreductase-like proteins (DsbA-L) in the ER. This is closely related to adipocyte ER stress and a maladaptive UPR [85] (Figure 2 and Figure 3). Adiponectin prevents the loss of telomerase activity and premature vascular senescence during hyperglycemia by activation of AMPK/TSC2/mTOR/S6K1 signaling and inhibition of PI3K/Akt/mTOR/S6K1 signaling [86]. Adiponectin protects against WAT metaflammation and senescence, pancreatic β-cell senescence, hepatic steatosis and fibrosis, Kupffer cell activation and liver cirrhosis/hepatocellular carcinoma, hepatocyte and renal podocyte senescence, and premature aging and death [85,87,88] (Figure 5).

## 5. Adipose Tissue Physiology and Energy Homeostasis

Normal adipose tissue plays an important endocrine role as a flexible energy depot and a regulator of energy homeostasis [89]. Adipose tissue comprises white adipose tissue (WAT), brown adipose tissue (BAT), and beige/brite adipose tissue [90]. White adipose tissue stores excess energy as triacylglycerides (TAG). Brown and beige AT maintain body temperature by increased mitochondrial density, uncoupling protein 1 (UCP-1) expression, and non-shivering (adaptive) thermogenesis via mitochondrial fatty acid β-oxidation. Heat is generated by UCP-1, dissipating the electrochemical proton gradient across the inner mitochondrial membrane and uncoupling oxidative phosphorylation and mitochondrial respiration. UCP-1 activity is enhanced by cold exposure, the stimuli of PPARγ agonists, sympathetic nerve activation of β1/β2/β3 adrenergic receptors (β-ARs), and T3 activation of thyroid hormone receptors (TRα-1, TRβ-1, TRβ-2) in BAT and beige adipocytes. T3 is permissive for noradrenaline-mediated non-shivering thermogenesis by regulating adrenergic sensitivity via TRα-1 signaling and upregulating *UCP-1*, *PGC1*α, and other mitochondrial metabolic machinery via TRβ activation. The presence of ROS or the release of long-chain fatty acids by sympathetic nervous system stimulation of lipolysis also increases UCP-1 activity. UCP-1 compensates for the loss of ATP production and decreased mitochondrial membrane potential during adaptive thermogenesis by increasing fatty acid β-oxidation [91]. 

### 5.1. WAT Distribution

WAT can be further divided into SAT (normally > 80% of TBF) and VAT (10–20% of TBF) [92,93]. Visceral fat is deposited in and around the internal organs of the abdominal and thoracic (epicardial, pericardial) cavities. Intra-abdominal VAT can be further classified into intraperitoneal viscera (omentum, liver, pancreas, intestinal mesentery) and extraperitoneal viscera (kidneys, rectum, uterus, and bladder). Intraperitoneal VAT with venous drainage into the portal vein is particularly metabolically active and involved in the release of adipokines, cytokines, exosomes, glycerol, and FFAs directly to the liver. In adult humans, BAT is found in the neck, mediastinum, and retroperitoneum, which maintain heat around the heart and great vessels [92,93]. 

### 5.2. WAT Physiology 

Normal WAT consists of differentiated adipocytes and the stromovascular fraction (SVF), including ADSC/adipocyte progenitors, endothelial cells, fibroblasts, T lymphocytes, and yolk-sac-derived macrophages [94]. Adipocyte number is determined in childhood and maintained in adults by apoptosis of mature adipocytes, as well as preadipocyte-/adipose-derived stem cell (ADSC) differentiation and maturation (adipogenesis). Adipocytes have a normal lifespan of approximately 8 years, and their replacement is controlled by transcription factors peroxisome proliferator-activated receptor-γ (PPARγ) and CCAAT/enhancer-binding protein-α (C/EBPα) [89]. The expression of PPARγ and C/EBPα is inhibited by transforming growth factor beta (TGF-β)-receptor I/activin receptor-like kinase (ALK4), which suppresses human preadipocyte differentiation and promotes adipose tissue hyperplasia [95]. Early WAT adipogenesis can also be suppressed by omega-3 (ω-3) polyunsaturated fatty acids (PUFAs), which may reduce the absolute number and differentiation of white adipocyte progenitors in the SVF without causing metabolic dysfunction [96].

On average, the percentage of total body fat (TBF) in adult women is 34% more than in men when adjusted for height. In premenopausal women, fat is typically deposited in superficial SAT of the gluteo-femoral areas under the influence of estrogen (gynoid fat mass distribution pattern) in preparation for reproduction and lactation. Males and post-menopausal women have more central fat deposition in the trunk and abdomen (android fat distribution). The mean VAT/TBF percentage in young males (15–18%) is double that of young females (7–8%) and increases with age in both sexes until 70 years of age. In young adults, fat is preferentially deposited in SAT until a threshold is reached of 23% TBF/TBW in males and 38% TBF/TBW in females, and then VAT mass increases significantly with increasing TBF percentage [97,98,99,100]. In population studies, subcutaneous fat mass is maintained in women until age 70, after which it dramatically declines, but the SAT mass decreases in a linear fashion in males after age 40. There is a steady increase in VAT% from 40 to 70 years in males and females, which continues to increase in females after 70. This is related to the fall in SAT volume in females after age 70 [97]. 

Post-prandial pancreatic insulin secretion is primarily induced by the elevation of plasma glucose and secondarily augmented by amino acids and free fatty acids [101,102,103,104]. Insulin promotes the PI3K/AKT/mTOR anabolic pathway, resulting in increased cellular glucose uptake and utilization, glycogen and protein synthesis, lipogenesis, and reduced lipolysis and hepatic gluconeogenesis. Nutrients are stored as WAT triglyceride, hepatic and muscle glycogen, cytosolic lipid droplets, or circulated as lipoproteins [101,102,103,104]. Very-low-density lipoproteins (vLDLs) produced from chylomicrons in the liver transport triglycerides to tissues, where endothelial lipoprotein lipases act on triglycerides to release free fatty acid (FFA) and glycerol. Adipocytes normally cannot utilize this glycerol as they lack glycerol kinase—they must instead manufacture glycerol-3-phosphate from glucose via glycolysis or from pyruvate via limited gluconeogenesis. Under insulin stimulation, FFAs are transported into the intracellular compartment by CD36 and fatty acid transport protein (FATP). Insulin increases membrane glucose uptake by adipocytes, cardiac, and skeletal muscle by rapidly increasing (5–30-fold) the availability of GLUT4 from intracellular storage pools. In adipocytes, glucose provides carbon backbones for glycerol synthesis and stimulates the lipogenic transcription factor: carbohydrate response element-binding protein (ChREBP). Non-esterified fatty acids (NEFAs) are esterified with glycerol backbones, forming TAG, and stored in a single large adipocyte lipid droplet [103]. 

### 5.3. WAT TAG Storage 

**Figure 6:**
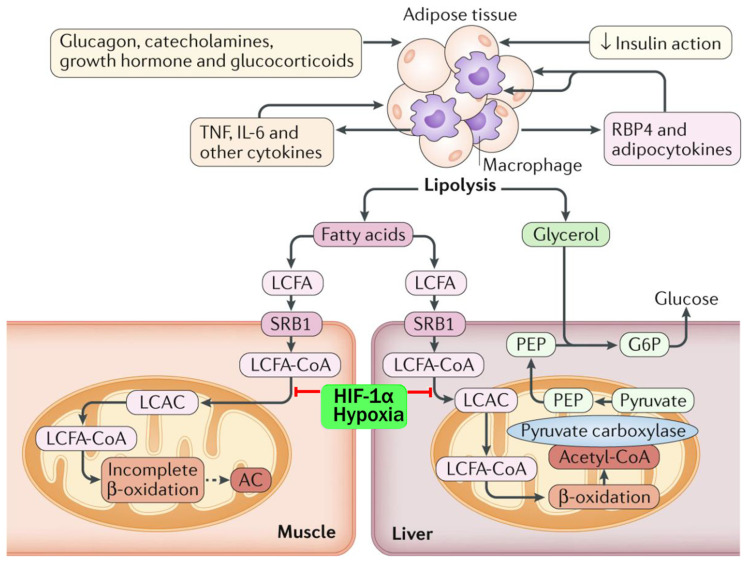
Alterations in lipid metabolism are associated with insulin-resistant states [108]. Obesity and T2DM are associated with increased lipolysis in adipose tissue owing to the action of or resistance to multiple hormones and to the increased production of cytokines (tumor necrosis factor (TNF) and interleukin-6 (IL-6)) by ATMφs. The release of TNF and IL-6 from macrophages is potentiated by the adipocyte secretion of adipocytokines, such as retinol-binding protein 4 (RBP4). FFAs, including long-chain fatty acids (LCFAs) that are released by lipolysis, are taken up by muscle and liver via the fatty acid transporter scavenger receptor class B member 1 (SRB1). In muscle, LCFA thioesters (LCFA-CoAs) are imported into mitochondria for β-oxidation via the carnitine shuttle, in which LCFA-CoAs are converted into long-chain acylcarnitines (LCACs). Incomplete β-oxidation in insulin-resistant states causes accumulation of acylcarnitines (ACs) of varying lengths, which are associated with insulin resistance and hyperglycemia. In the liver, LCFAs are imported into mitochondria and oxidized to generate acetyl-CoA, which activates pyruvate carboxylase, leading to increased production of phosphoenolpyruvate (PEP) from pyruvate. Glycerol generated from lipolysis, in addition to PEP, is converted into glucose-6-phosphate (G6P), resulting in increased hepatic glucose production. Overall, the increased flux of metabolic substrates into liver causes insulin resistance and hyperglycemia. Although an increase in AC muscle content correlates with insulin resistance, a causative effect has not been established in vivo. Hypoxia and stabilization of HIF-1α blocks mitochondrial fatty acid β-oxidation by inhibiting LCAD, MCAD, and CPT1 and leads to lipotoxicity and insulin resistance. Reproduced from Yang et al. (2018) [108].

The storage of triglycerides in SAT is achieved by adipocyte hyperplasia (proliferation and differentiation of preadipocytes) and hypertrophy, allowing expansion of WAT in response to excess energy balance [54,105,106,107]. VAT tends to have fewer preadipocytes and is prone to develop adipocyte hypertrophy and metaflammation [89]. Insulin promotes lipid storage by activating SREBP1c, ACC, DGAT, and FAS, which are negatively regulated by adrenaline, noradrenaline, and glucagon. Fatty acid synthase (FAS) catalyzes de novo synthesis of long-chain saturated FAs (palmitate) from acetyl-CoA, malonyl-CoA, and NADPH; glycerol-3-phosphate acyltransferase (GPAT-3) converts glycerol-3-phosphate and long-chain acyl-CoA to lysophosphatidic acid; and diacylglycerol acyltransferase (DGAT) catalyzes TAG esterification. This is required for de novo lipogenesis and storage of TAG [54,105,106,107]. Elevated glucose levels stimulate ChREBP via the non-oxidative pentose phosphate pathway (PPP), which promotes lipogenesis and glycolysis genes (liver pyruvate kinase, ACC, FASN). Acetyl-CoA carboxylase (ACC) is the rate-limiting step in FFA synthesis, determining the fate of acetyl-CoA and preventing simultaneous lipogenesis and FAO. ACC catalyzes the conversion of acetyl-CoA to malonyl Co-A, which inhibits carnitine-palmitoyl transferase 1 (CPT1) and promotes lipogenesis. Phosphorylation of ACC (pACC) inhibits its activity, which promotes FAO [108] (Figure 6). During the development of obesity, systemic levels of cortisol are low or normal, but there is increased local synthesis of cortisol in humans due to 11-beta hydroxysteroid dehydrogenase 1 (HSD1) activity in WAT. Cortisol tends to have pleiotropic effects in different lipid depots and may contribute to central obesity due to adipocyte hypertrophy in VAT. Cortisol decreases lipogenesis and FFA uptake in basal or fasting conditions but acts synergistically with insulin to upregulate lipogenesis [109,110].

### 5.4. WAT Lipolysis

In response to negative energy balance, hydrolyzation of triglycerides stored in WAT releases FFAs for β-oxidation in the target tissue and glycerol for hepatic gluconeogenesis to meet the metabolic demand [108]. Stimulation of TAG lipolysis during fasting, cold exposure, or moderate aerobic exercise (45–65% VO_2_max) is mainly via catecholamine activation of G-stimulatory protein (G_s_)-coupled β-adrenergic receptors (β-ARs) on the adipocyte plasma membrane. This activates adenylyl cyclase, which catalyzes the formation of 3′,5′-cyclic adenosine monophosphate (cAMP) from ATP. The rise in intracellular [cAMP] removes the inhibition of cAMP-sensitive protein kinase A (PKA). PKA promotes phosphorylation of adipocyte triglyceride lipase (ATGL) and hormone-sensitive lipase (HSL). Access of these lipases to the lipid droplet is controlled by perilipin (PLIN), which is also phosphorylated by PKA. Triglyceride is hydrolyzed by ATGL to form diacylglyceride (DAG), which is then hydrolyzed by HSL to monoacylglyceride and then to glycerol and a NEFA by monoacylglycerol lipase (MAGL). Glycerol is released into the bloodstream via aquaporin 7 for use in hepatic gluconeogenesis. NEFA can be released and transported bound to albumin for oxidation in skeletal muscle or returned to the liver for oxidation, production of acetyl CoA, or re-esterification and export as vLDL. Conversely, the formation of cAMP by adenylyl cyclase and downstream TAG lipolysis in human adipocytes is inhibited by membrane α_2_-adrenoceptors (α_2_-ARs), which are coupled to G-inhibitory proteins (G_i_). Catecholamines have a higher affinity for α_2_-ARs than β-ARs, and in human adipocytes, α_2_-ARs outnumber β-ARs. Thus, under low levels of catecholamines in humans, lipolysis is normally inhibited. The expression of adrenergic receptors is reversed in murine adipocytes, where the expression of β-ARs is normally higher than for α_2_-ARs. During human obesity, there is an increase in α2-Ars and α2/β-AR ratios and α2-AR-mediated responses, which contributes to the impairment of catecholamine-stimulated lipolysis [111].

Adipocyte lipolysis is also increased by cortisol, glucagon, T3, atrial/brain natriuretic peptide, and growth hormone and is opposed by insulin [69,111,112,113,114]. Insulin signaling inhibits PKA by activating phosphodiesterase 3b (PDE3B), which hydrolyzes cAMP, and also by inhibiting PKA locally at the lipid droplet through AKT-independent but PI3K-dependent pathways. Insulin decreases ATGL transcription via mTORC1 or FOXO1 signaling. Multiple ligands can also activate Gi-coupled receptors to inhibit lipolysis, including fatty acids, lactate, succinate, ketone body β-hydroxybutyrate (β-OHB), nicotinic acid, and neuropeptide Y. Local accumulation of NEFA thus inhibits HSL activity, which is why oxidation or export of mobilized NEFA is required for lipolysis to continue in adipocytes [69,111,112,113,114]. 

Medium-chain (C6–C12) and long-chain (C13–C20) fatty acids are converted to acyl-CoAs by acetyl-CoA synthetase in the cytoplasm, and then long-chain fatty acyl-CoAs (LCFA-CoA) are converted to long-chain acyl-carnitines (LCAC) by CPT1 for transport into the mitochondria via the carnitine shuttle [102]. Medium-chain fatty acyl-CoAs do not require special transporters to cross mitochondrial membranes. Medium- and long-chain fatty acyl-CoAs undergo β-oxidation in the mitochondria, each cycle of which removes two carbons and produces one molecule of Acetyl-CoA, FADH2, and NADH. Acetyl-CoA is used in the tricarboxylic acid cycle, and FADH2 and NADH are sent to the electron transport chain to generate ATP. β-oxidation of very-long-chain acyl fatty acids (>20 carbon chains) occurs in the peroxisome [103] (Figure 6).

### 5.5. WAT, Adiponectin, AMPK, and Autophagy

Healthy WAT in lean individuals is well vascularized with normal capillary density. It contains mainly small (<50 μm) and medium-size (50–69 μm) adipocytes that normally secrete anti-inflammatory adipokines (adiponectin, C1q/TNF-related protein (CTRP) family, omentin-1, fibroblast growth factor 21 (FGF21), secreted frizzled-related protein 5 (SFRP5)), which modulate systemic energy homeostasis [115,116]. Noradrenaline and ATP are released from sympathetic nerve endings in adipose tissue when the sympathetic nervous system is activated [115]. Noradrenaline binding of adipocyte β3-ARs activates *e*xchange *p*roteins *a*ctivated by *c*AMP, isoform 1 (Epac1), and ATP activation of adipocyte purinergic (P2Y2) receptors increases intracellular Ca^2+^ ([Ca^2+^]*i*). These act synergistically to potently increase exocytosis of vesicles containing HMW adiponectin from adipocytes [115]. During obesity, adipocyte expression of β3-ARs is decreased by >50% and Epac1 by >70% compared to lean adipocytes, contributing to a decrease in HMW adiponectin release [115].

Adiponectin constrains metaflammation by promoting IL-10 release, M2 macrophage polarization, and oxidative phosphorylation and inhibiting the release of inflammatory cytokines, including TNF-α and IFN-γ [116]. Normal plasma adiponectin concentrations range from 5 to 30 μg/mL, with the HMW adiponectin multimer being the most physiologically active form [117]. Adiponectin is particularly important in maintaining lean body weight and preventing hepatic steatosis by activation of its cognate membrane receptors AdipoR1 and AdipoR2, both of which are ubiquitously expressed. However, AdipoR1 is the major adiponectin receptor expressed in skeletal and cardiac muscle, macrophages, and the hypothalamus. AdipoR1 agonism activates AMPK, insulin receptor substrate (IRS)-1 and IRS-2 via adaptor protein, phosphotyrosine interaction, PH domain, and leucine zipper containing 1 (APPL1) and phosphorylates p38 MAPK pathways. This enhances FAO (pACC), vasodilation (eNOS), and insulin sensitivity (GLUT-4) and inhibits inflammatory NF-κB pathways and anabolic mTORC1 pathways [118]. Under starvation conditions, glucose deprivation, or a decrease in the cellular ATP/AMP ratio, AMPK release is stimulated. AMPK promotes the inhibitory effects of tuberous sclerosis proteins 1 and 2 (TSC1/2) on mTORC1, which enhances autophagy and lipophagy. This promotes *catabolism* of energy stores and restores ATP production by mitochondrial biogenesis, glucose utilization, glycogenolysis, lipolysis, and lipid oxidation and suppresses mTORC1 anabolic processes, including lipogenesis, glycogen storage, gluconeogenesis, and protein synthesis. However, under excessive nutrient conditions (glucose, palmitate), mTORC1 is activated, adiponectin secretion and activity of AMPK and SIRT-1 is impaired, and ROS formation is enhanced. This inhibits the fusion of autophagosomes and lysosomes, lysosomal function (lysosomal acid lipase, acidification, protease activity), and autophagy. Impaired autophagy can result in dysfunctional substrate clearance, intracellular retention of the senescence regulator GATA4/p62, lipid droplet accumulation, lipid peroxidation and further mitochondrial dysfunction and ROS release, lysosomal membrane instability and release of incompletely degraded toxic substrates, activation of DAMP receptors, and promotion of cellular senescence [29,69,119,120].

**Figure 7:**
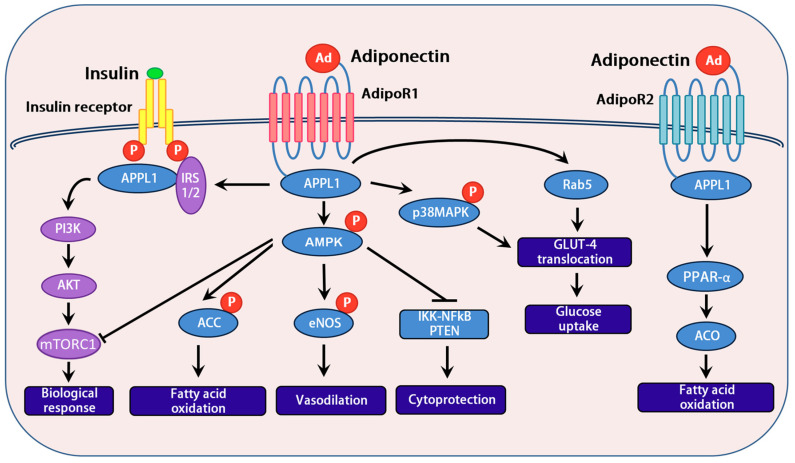
Schematic representation of adiponectin signal transduction [118]. Implicating crosstalk with the insulin signaling pathway: insulin and adiponectin interact with their respective receptors, which trigger a cascade of signaling events. Insulin actions are mainly carried out by PI3K/AKT pathway, resulting in increased protein synthesis, lipogenesis, glucose uptake and utilization, glycogen synthesis, and reduced lipolysis and gluconeogenesis. Interaction of adiponectin with its receptors (Adipo R1 and R2) results in the activation of multiple signaling pathways, including IRS1/2, AMPK, and p38 MAPK. Activation of IRS1/2 by adiponectin signaling is a major mechanism by which adiponectin sensitizes insulin action in insulin-responsive tissues. Adiponectin drastically increases the expression and activity of PPARα, which upregulates acetyl-CoA oxidase (ACO), CPT1, and uncoupling proteins (UCPs), promoting FAO and energy expenditure. Reproduced from Achari et al. (2017) [118].

Loss of heat shock response also inhibits the protective effect of adiponectin, as heat shock protein 60 (HSP60) modulates adiponectin signaling by stabilizing AdipoR1 and AdipoR2 [84] (Figure 5). AdipoR2 is highly expressed in the liver, and its agonism by adiponectin greatly enhances hepatic PPARα expression and activity, which increases mitochondrial FAO via acetyl-CoA oxidase (ACO), CPT1, and UCPs [118] (Figure 7).

## 6. WAT Senescence, SASP, and Lipotoxicity

In diet-induced obesity, when the threshold of adaptive response to excessive energy intake is reached, further SAT storage expansion by adipocyte hypertrophy is limited, and adipocyte hyperplasia is inhibited by pre-adipocyte senescence. This results in loss of both AT remodeling and WAT lipid buffering capacity, with increased FFA circulation and TAG deposition in VAT and ectopic sites—the *adipose tissue expandability hypothesis* [89,121]. Adiponectin secretion is inversely related to increasing BMI, WHR, and VAT volume, and loss of HMW adiponectin greatly inhibits adipogenesis and SAT storage expandability [122]. 

Hypoxia in hypertrophied adipocytes interferes with ascorbate and oxygen-dependent disulfide bonding and protein folding in the ER lumen [85,88]. This results in adipocyte ER stress, a maladaptive UPR, and increased ER stress markers, including CHOP and BiP/GRP78. In an in vitro model of obesity-associated cellular stress, exposure of preadipocytes and adipocytes to palmitate and TNF-α significantly increased markers of hypoxia (HIF-α, GLUT1, VEGF), ER stress (sXBP1, CHOP, BiP/GRP78), and inflammation (IL-6, MCP-1, and TNF-α) [85,88]. WAT hypoxia progresses once the size of hypertrophic adipocytes exceeds the diffusion limit of oxygen (>100 μm). Inflammation, hypoxia, altered adipokine secretion profile, and fibrosis all contribute to cellular stress in hypertrophied WAT [123,124]. Persistent ischemia in VAT can be worsened by oxidative stress and OSA in obesity [125]. Hypoxia due to impaired vascularization, decreased capillary density, and defective neoangiogenesis; ROS generation; oxidation products (e.g., oxidized LDL); pyruvate excess; or HFD intake can also lead to WAT mitochondrial oxidative stress, ER stress, unresolved UPR, and SASP (Figure 3 and Figure 5).

Adipocyte UPR/SASP increases the release of pro-inflammatory adipokines and decreases the release of anti-inflammatory adipokines (adiponectin) [103,126]. Increased secretion of inflammatory adipokines leptin, resistin, visfatin, osteopontin, angiotensin II, IL-1β, IL-6, and TNF-α by hypertrophic adipocytes leads to excessive ROS generation and decreased antioxidant capacity. This furthers WAT oxidative stress, NF-κB-mediated inflammation, and recruitment of adipose tissue macrophages (ATMφs) [127]. Prolonged or excessive ER stress can induce either cell apoptosis via autophagy and mitophagy or cellular senescence. Cellular senescence is closely related to an impaired heat shock response to ongoing inflammation and downregulated HSP72, particularly during persistent NF-κB activation [57]. Pro-inflammatory cytokines can also increase pathological lipid/NEFA release, especially from obese VAT. Such impaired lipid storage in WAT contributes to ectopic deposition of lipids in non-adipose tissues, including skeletal muscle, liver, kidneys, heart, and pancreas. This is referred to as lipotoxicity [89,103]. Adipocyte SASP can have an autocrine effect by promoting senescence in already senescent adipocytes, a paracrine effect of promoting senescence in surrounding cells (ATMφ, ADSC, WAT stromal vascular fraction), and endocrine senescent effects on distant organs (muscle, heart, pancreas, liver, kidneys, vascular endothelium, hypothalamus) [128] (Figure 5).

## 7. Extracellular Vesicles and Cellular Senescence

Extracellular vesicles (EVs) are nanosized membrane vesicles important for intercellular communication [129,130,131,132,133,134,135,136,137,138,139]. EVs are secreted by all cells and found in most biological fluids. During cellular senescence, EVs are a key component of the SASP. WAT is the largest endocrine organ and can secrete large numbers of EVs, with differentiated white adipocytes producing the highest numbers of EVs. WAT is a major source of circulating exosomal miRNA in vivo, which regulates gene expression in other tissues [140,141]. Co-culturing of normal ADSCs with hypertrophic SAT adipocytes from HFD diet-induced obese male C57BL/6 mice was able to achieve the following:

- Transfer ER stress to ADSCs with the development of a significantly elevated expression of ER stress markers *Xbp1*, *sXbp1*, *Atf4*, *Atf6*, and *Grp78* in ADSCs;
- Induce senescence in ADSC with significant increases in mRNA expression and protein levels of P16 and P21 and SASP (IL6 and Ccl2);
- Decrease ADSC adipogenic differentiation potential (*Pparg*, *Plin1*, and *Insr* expression);
- Decrease ADSC telomere length and increase population doubling time and positive rate of SA-β-gal.

These senescent effects were mediated by EVs released from senescent mature adipocytes and taken up by ADSCs. The inhibition of exosome production through the administration of the sphingomyelinase inhibitor GW4869 abrogated these effects. This demonstrated the role of EVs in spreading ER stress and cellular senescence in hypertrophic obesity [52]. 

### 7.1. EV Biosynthesis and Cargo

**Figure 8:**
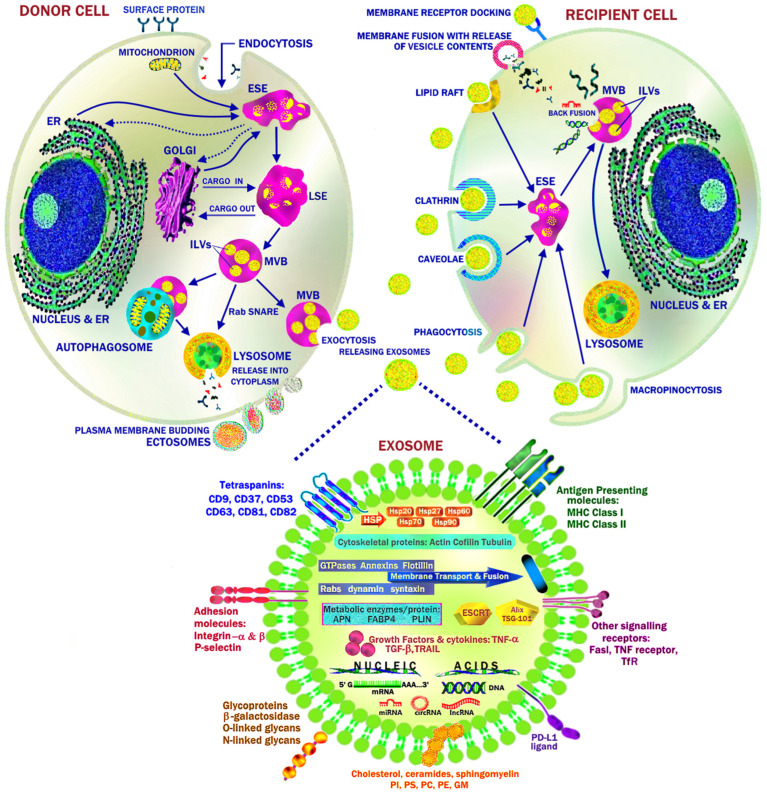
Biogenesis and release of exosomes and recognition and endocytosis by recipient cell. The transfer of multivesicular bodies (MVBs) to lysosomes for degradation (autophagy) or their fusion with the plasma membrane and ILV exocytosis as exosomes (which is increased in cellular senescence) is depicted. The enlarged exosome diagram shows exosome cargo and membrane molecules and receptors. The formation of microvesicles by outward plasma membrane budding (ectosomes) is also shown. Exosomal membrane lipids include cholesterol, ceramides, sphingomyelin, phosphatidylinositol (PI), phosphatidylserine (PS), phosphatidylcholine (PC), phosphatidylethanolamine (PE), and gangliosides (GM) [133,135,139].

Extracellular vesicles can be broadly classified into ectosomes and exosomes [129,130,131,132,133,134,135,136,137,138,139]. Formation of exosomes involves the invagination of the donor cell plasma membrane with cell surface proteins (endocytosis) and the generation of an early sorting endosome (ESE) with interactions with mitochondria, the nucleus, the endoplasmic reticulum, and Golgi apparatus. A late sorting endosome (LSE) is then formed with further cargo loaded from the Golgi and cytoplasm, including nucleotides, ncRNA, and soluble proteins. Invagination of the limiting membrane of multivesicular bodies (MVBs) creates intraluminal vesicles (ILVs). ILVs have a diameter of 40–160 nm and are ultimately secreted by exocytosis as exosomes. This occurs via the transport of MVBs by cytoskeletal actin and microtubule networks and motor proteins such as kinesin and dynein to the plasma membrane, with which it fuses under the direction of Rab GTPases and soluble N-ethylmaleimide-sensitive factor attachment protein receptor (SNARE). The exosomes are then released into the extracellular space [129,130,131,132,133,134,135,136,137,138,139]. EVs can also be secreted as ectosomes by budding from the plasma membrane as microvesicles (100–500 nm) or from apoptotic cells as apoptotic bodies (50–5000 nm) by membrane blebbing [129,130,131,132,133,134,135,136,137,138,139] (Figure 8).

ILV generation in the LSE/MVBs can be achieved by three major mechanisms:

- Classical pathway. The endosomal-sorting complex required for transport (ESCRT). There are four different ESCRT subcomplex proteins (ESCRT-0, -I, -II, -III) that interact with ATPase vacuolar protein sorting-4 (VPS-4) to sequentially coordinate the formation of ILVs inside MVBs [134,142]. ESCRT-0 and -I have ubiquitin-binding domains that can capture, sort, and load mono-ubiquitinated proteins into MVB compartments. Ubiquitin-binding ESCRT subunits include hepatocyte growth factor-regulated tyrosine kinase substrate (Hrs), Signal-Transducing Adaptor Molecule (STAM1) in ESCRT-0, and tumor susceptibility gene 101 (TSG101) in ESCRT-1 [129,130,131,132,133,134,135,136,137,138,139]. Examples of ubiquitinated proteins incorporated into ILVs include antigen-presenting molecules (MHC I and II), epidermal growth factor receptors (EGFRs), and ligand–receptor signaling complexes. When TSG101 interacts with ESCRT-1, it results in the recruitment of ESCRT-II. This begins the deformation of the endosomal limiting membrane and internal budding around collections of ubiquitinated proteins and the interaction between the charged multivesicular body protein-6 (CHMP6) subunit of ESCRT III and ESCRT II. The polymerization of flat spiral polymers into helical filaments by the ESCRT-III complex facilitates vesicle budding, membrane protrusion, and neck compression. Hydrolysis of ATP by AAA-ATPase VPS-4 then provides the energy for the sequential removal of ESCRT helical filaments and scission of the vesicle from the endosome membrane. Exosome production can be increased by leptin, as it stimulates increased expression of TSG101 [130,138,143,144].
- ALIX (apoptosis-linked gene 2-interacting protein X)/syndecan/syntenin pathway. This pathway is activated by Src kinase phosphorylation of ALIX, syndecan1, and syntenin. Src kinase is inhibited by the Src tyrosine kinase inhibitor dasatinib, which inhibits mTOR, promotes autophagy, and dramatically reduces exosome release [145,146,147,148]. Activation of phospholipase D2 (PLD2) by the GTPase ADP-ribosylation factor 6 (ARF6) enables the recruitment of syntenin to the endosome membrane. There, it recruits ALIX, which then associates with ESCRT III via lysobisphosphatidic acid (LBPA). Interactions between ALIX and ESCRT-III facilitate cargo sorting and membrane budding. This is independent of early ESCRT machinery but still requires VPS-4 for ILV scission [130,138,143,144].
- Ceramide pathway. The production of EVs can also occur independently of ESCRT pathways and involves the interactions between lipid rafts, tetraspanins, and proteins. Localization of neutral sphingomyelinase (nSMase2) to the endosome limiting membrane is required for the production of ceramide lipid rafts from sphingomyelin, and, together with Rab31 and flotillin, it promotes its initial curvature and inward budding for the formation of ILVs. This process can be blocked by the sphingomyelinase inhibitor GW4869 or inhibition of cholesterol synthesis with 3-hydroxy-3-methyl glutaryl coenzyme A reductase inhibitors such as simvastatin or lovastatin. Molecules associated with ceramide lipid raft microdomains include lipids (cholesterol, ceramide, sphingomyelin, glycosphingolipid, and ganglioside), tetraspanins (CD9, CD63, and CD81), flotillin, glycosylphosphatidylinositol (GPI)-anchored proteins, membrane tyrosine kinase receptors (EGFR, PDGFR), and heat shock proteins. The tetraspanin CD63/LAMP3 is particularly important in the sorting and loading of MVB cargo, as well as the direction of CD63^+^ MVBs toward exocytosis rather than autophagy. This is due to the phosphorylation of Rab 31 by EGFR, which leads to the recruitment of flotillin proteins and the further insertion of EGFR (and other TKRs: IGF1R, MET, PDGFR-β, FGFR2) onto the lipid microdomains of CD63^+^ MVBs [130,138,147,149].

ALIX facilitates the sorting and insertion of tetraspanins and G-protein-coupled receptors (GPCRs) into the late endosomal membrane by ubiquitination-independent recruitment of ESCRT III [130,138,142]. In contrast, ALIX is also involved in the trafficking of PD-L1 to the endosomal membrane under the influence of phosphorylation of Vps27/Hrs by extracellular signal-regulated kinases (ERKs) [147]. The presence (tetraspanin-6) or absence (CD63) of tetraspanins can determine the fate of MVBs by their direction to lysosomes and ILV degradation (autophagy) [136]. During nutrient excess, mTORC1 can inhibit the initiation of autophagy by phosphorylating unc-51-like kinase 1 (ULK1) and autophagy-related gene 13 (ATG13) but also inhibit the fusion of the autophagosome with the lysosome by regulation of Rab7 and vATPase. Whilst the Rab GTPases Rab2b, Rab5a, Rab9a, Rab27, Rab31, and RAL-1 promote MVB fusion with the plasma membrane and exosome release, Rab7 promotes transport and fusion of MVBs with lysosomes for degradation. Rab7 activity is indeed inhibited by Rab 31 and increased by AMPK [25,119,120,130,147,150]. ATG5 and ATM both act to increase the pH of autophagosomes by regulating vATPase, thereby promoting the trafficking of MVBs to the plasma membrane for exocytosis [130,138,147,149].

Exosomes contain the constituents of their parent cells, but these may be enriched or depleted in different physiological states and contexts, including exercise-trained versus sedentary individuals, inflammation and senescence, youth and age, and lean and obese individuals [129,130,131,132,133,134,135,136,137,138,139]. Exosomal cargo and membrane-bound molecules include the following:

- MHC-presenting molecules (MHC class I, MHC class II);
- Receptor ligands (integrin-α, integrin-β, P-selectin, PD-L1);
- Tetraspanin proteins (CD9, CD37, CD53, CD63, CD81, CD82);
- Other surface signaling proteins (Fas ligand (FasL), TNF-R, transferrin receptor (TfR));
- Heat shock proteins (HSP 20, HSP27, HSP60, HSP70, Hsc70);
- Cytoskeletal proteins (actin, myosin, cofilin-1, tubulin, vimentin, fibronectin);
- Membrane transport and fusion proteins (GTPases, annexins, flotillins, Ras-associated binding (Rab) proteins, dynamin, syntaxin);
- Proteins involved in the biogenesis of exosomes (TSG101, ALIX, ESCRT complex);
- Growth factors and oncogenic signaling proteins (TNF-α, TGF-β, TRAIL, PI3K, HIF-1α, EGFR, β-catenin, KRAS);
- Metabolic enzymes involved in glycolysis and lipogenesis (ATPase, PGK, PK, GAPDH, enolase; ACC, glucose-6-phosphate dehydrogenase (G6PD), FAS);
- Glycoproteins (β-galactosidase, O-linked glycans, N-linked glycans);
- Lipid-associated proteins and phospholipases;
- Lipids and fatty acids (sphingomyelin, sphingosine 1-phosphate, phosphatidylserine, cholesterol, TAG, palmitic acid, ceramides);
- Adipokines (adiponectin);
- Ferritin carrying iron [18];
- Nucleic acids (mRNA, miRNA, lncRNA, circRNA, mtRNA, tRNA, DNA, mtDNA) [139];
- Organelles, including mitochondria [129,130,131,132,133,134,135,136,137,138,139] (Figure 8).

After being transcribed from nuclear DNA, primary miRNA (pri-miRNA) is processed by the nuclear RNase III Drosha into precursor RNA (pre-miRNA) [151]. Hairpin pre-miRNA is then transported to the cytoplasm via Exportin 5, where it is cleaved by the RNase DICER/double-stranded RNA-binding protein (TRBP) complex to form 21–22 nucleotide double-stranded miRNA [151]. Each of the 5p and 3p miRNA strands is taken up by the Argonaute proteins (AGO1-4) in the miRNA-induced silencing complex (miRISC), which mediates translational suppression of its target mRNA (by miR-AGO1) or degradation of its target mRNA (by miR-AGO2) with the assistance of glycine–tryptophan protein of 182 kDa (GW182) [152]. The loading of miRNA from the cytoplasm into developing MVBs can be facilitated by one of four mechanisms:

- The miRISC-associated pathway;
- The neutral sphingomyelinase 2-dependent (nSMase2) pathway;
- RNA-binding protein-dependent pathway (heterogeneous nuclear ribonucleoproteins (hnRNPA2B1) and synaptotagmin-binding cytoplasmic RNA-interacting protein (SYNCRIP));
- The miRNA sequence-dependent pathway [153,154].

### 7.2. Exosomes and Epigenetic Control of Gene Expression

Epigenetic control of DNA gene expression includes exosomal transfer of ncRNA. Both mRNA and ncRNAs are transcribed and processed, but ncRNAs (miRNA, circRNA, lncRNA) do not produce proteins [131]. MicroRNAs regulate protein production by binding to 3′ untranslated regions (UTRs) of their target mRNAs and inhibiting the translation of specific RNAs inside the miRISC complex [129,132,135]. Exosomes are enclosed in a lipid bilayer, making them stable in serum and protecting their RNA cargo, which would otherwise be degraded by RNases in bodily fluids and tissues or generate an immune DAMP response to extracellular nucleic acids. MicroRNA can also be protected and transported by AGO2 proteins or high-density lipoproteins (HDL)/LDLs in the circulation [155,156]. More than 60% of gene expression can be regulated by ncRNA at the stages of transcription, post-transcription, or translation, and ncRNA is widely distributed by exosomes [137]. The exosomal mRNA can also be transferred intact into the recipient cell and undergo translation to functional proteins [157]. The components of the miRISC complex pathway (AGO2, GW182, YBX1 (Y-box protein I)) have been found in exosomes or colocalized with MVBs. Whether the entire miRISC complex is transferred via exosomes is controversial; however, the exosomal miRNA can utilize recipient cell RISC to degrade or inhibit their corresponding mRNA [157]. 

Exosomes secreted by different cells may have heterogeneous sizes, cargos, transmembrane proteins (tetraspanins), MHC-presenting antigens, adhesion molecules (integrins), glycoproteins, lipids, and other surface signaling receptors and ligands, which determine their subsequent recognition by specific target cells [130,138]. This can occur via exosomal docking with surface protein receptors/ligands, including immunoglobulins, ephrins, integrins, or tetraspanins, and enable receptor-activated signaling or direct interaction with recipient cells [158]. Docking is followed by secure membrane fusion with target cell plasma membranes, formation of a fusion pore via SNARE complexes, and release of the exosome contents into the recipient cell cytoplasm [158]. Exosomes can also be internalized by endocytosis, macropinocytosis, or phagocytosis. Endocytosis of exosomes can be mediated by clathrin, lipid rafts, or caveolin and result in the formation of an ESE and then an MVB, with subsequent degradation by lysosomes in the recipient cell [130,138]. Donor cells that are under stress can transfer exosomal cargo, including cytokines, growth factors, and DAMPs, which can thus be delivered to the target cell cytoplasm and generate an NF-κB inflammatory response. They may also translocate cell membrane attached- and spanning-proteins, which can be fused or backloaded from endocytosed MVBs and provide activated cell membrane surface receptors. This includes antigenic proteins and MHC proteins, as well as their intracellular signal transduction adapter proteins, such as MyD88, IRAK4, TRAF2, and TRADD [159] (Figure 8).

Exosomes contain the molecular effectors and machinery that profoundly influence metabolic health, body phenotype, appetite, foraging behavior, and energy regulation. Many of the effects of exosomal miRNAs/ncRNA are related to the promotion or inhibition of nuclear transcription factors and signaling pathways involving inflammation, insulin receptors, adipogenesis, ER stress, FAO, thermoregulation, macrophage polarization, glycolipid metabolism, and senescence, which are summarized in Table 1 [137,158,160,161,162,163,164,165,166,167,168,169,170,171,172,173,174,175,176,177,178,179,180,181,182,183].

### 7.3. Adipocyte-Derived Exosomes

**Figure 9:**
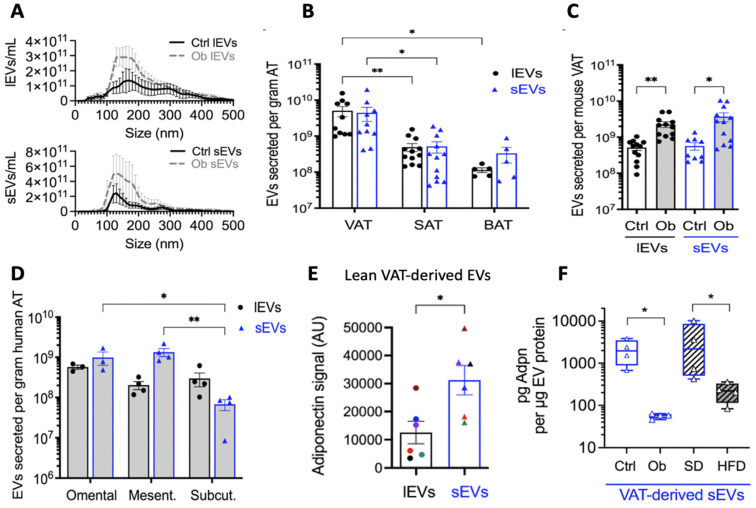
Exosome number and size, adiponectin secretion, and relationship to fat depot source and obesity. (**A**) Lean (Ctrl) and obese (Ob) mouse VAT-derived large extracellular vesicles (lEVs) and small extracellular vesicles (sEVs) mode size distribution curve comparison. Ctrl and Ob EV subtype size distribution curves are represented by plain or dashed lines, respectively, and are presented as the mean ± SEM. (**B**) lEV and sEV secretion from *ob*/*ob* mouse obese VAT, SAT, and BAT explants. EV number secreted per gram of AT per 24 h is presented. (**C**) Increased lEV and sEV secretion from *ob*/*ob* mouse VAT with obesity. EV secreted by total mouse VAT is presented. (**D**) EV secretion of human omental, mesenteric (Mesent.), and subcutaneous (Subcut.) AT collected from human subjects with obesity. Secretion of EVs is presented as the number of EVs secreted per gram of human AT. (**E**) VAT-derived lEVs and sEVs (8 mg each) from lean mice reveal a particular enrichment of adiponectin in sEVs. Each color represents an independent experiment, with lEV and sEV preparations derived from the same VAT. *n* = 6, mean ± SEM, * *p* ≤ 0.05 (t test for matched pairs). (**F**) ELISA quantification of the adiponectin content of VAT-derived sEVs isolated from lean or obese VAT explants. Obese VAT-derived sEVs, isolated from either *ob*/*ob* (Ob) or HFD mice, are depleted of adiponectin (Adpn) compared to sEVs produced from VAT collected from their respective lean control mice (Ctrl and SD). The results are presented as box and whisker plots with the mean from four sEV-independent samples measured for each condition (Ctrl, Ob, SD, and HFD). Data are presented as the mean ± SEM for (**A**–**F**), with dot plots representing independent samples. Statistical significance is indicated for each panel as follows: * *p* ≤ 0.05 and ** *p* ≤ 0.01. Reproduced from Blandin et al. (2023) [83].

Adipose tissue secretes EVs rich in functional lipids, RNAs, and proteins, leading to different paracrine and endocrine effects in target tissues [140]. WAT-derived exosomes are characterized by the presence of fatty acid-binding protein 4 (FABP4), perilipin-1, adiponectin, or PPAR proteins [185]. However, these proteins can vary greatly, depending on the degree of adipocyte differentiation or hypertrophy, and are also expressed by ATMφ. The majority of circulating exosomal miRNAs are derived from adipose tissue, particularly during obesity [186,187]. Adipose tissue-derived exosomes can thus exert epigenetic control over cellular metabolism, proliferation, differentiation, and behavior, as well as substrate utilization and storage in the body. In obese humans and experimental animal models using leptin-deficient mutant (*ob*/*ob*) or HFD-fed male C57BL/6 (B6) mice, the VAT depot is the highest secretor of EVs as compared to SAT and BAT. During obesity, increased EV secretion from VAT and SAT occurs, whereas EV production from lean BAT and obese BAT is similar. In tissue biopsies from patients with obesity undergoing bariatric surgery, VAT (omentum and mesentery) secreted significantly higher numbers of small EVs (exosomes) per gram of fat in comparison to that secreted by SAT [83] (Figure 9).

### 7.4. Regulation of Adipocyte EV Release

The secretion of EVs is regulated by both physiological and pathological mechanisms, which play an important role in energy and nutrient homeostasis. Adipocyte hypertrophy in obesity results in WAT inflammation, hypoxia, cellular senescence, and extracellular acidosis, as well as the accumulation of peroxides, ceramide, and saturated fatty acids (palmitate), all of which increase WAT exosome release [188,189,190]. Adipose-derived stem cells secrete miR-125a, miR-132, and miR-21, which promote angiogenesis in response to WAT hypertrophy and associated hypoxia [158,165,191]. A 10-fold increase in circulating plasma EVs was observed in obese individuals compared to lean counterparts, which correlated with increased homeostatic model assessment for insulin resistance (HOMA-IR) [192,193]. SIRT-1 expression in adipose tissue and circulating BMDM is significantly suppressed in human obesity, contributing to an increased release of AT-derived exosomes [194]. 

In contrast, lean WAT in healthy-weight individuals produces AMPK-α kinase and SIRT-1, which suppresses adipocyte exosome release [195]. During hypertrophic obesity in *ob***/***ob* mutant mice or HFD mice, HuR protein levels in SAT, WAT, and BAT fell due to decreased *HuR* mRNA expression. Decreased HuR leads to inhibition of autophagosome formation and loss of autophagy and ATGL function during adipocyte ER stress, contributing to cellular senescence and lipid retention. Intact HuR normally activates SIRT-1 and HSF-1, as well as autophagy-related gene (ATG) products ATG5, ATG12, and ATG16 [56,57,196,197,198] (Figure 4). Autophagy typically inhibits exosome release by ATG proteins interacting with ESCRT ALIX and mediating autophagolysosomal degradation of MVBs instead of fusion with the plasma membrane and exocytosis [136] (Figure 8). Knockdown of adipocyte-specific SIRT-1 in mice resulted in inhibition of autophagy, increased fat mass, adipocyte and hepatic insulin resistance, and hepatic gluconeogenesis/lipogenesis [194]. Loss of SIRT-1 also increased adipocyte lipolysis and impaired adipocyte fatty acid β-oxidation, resulting in elevated plasma glycerol and hypernefemia. This effect was directly related to the increased release of adipocyte-derived exosomes, exosomal activation of the TLR4/NF-κB signaling pathway, and phosphorylation of IRS-1 and AKT. Inhibition of exosome release by treatment with glutathione or GW4869 ameliorated these metabolic effects in Ad-*Sirt1*^−/−^ mice. Restoration of autophagy with rapamycin treatment (mTOR inhibitor) also reversed the increased exosome secretion in primary Ad-*Sirt1*^−/−^adipocytes [194].

### 7.5. Changes in Adipocyte-Derived Exosome Cargo

Changes in exosome cargo contents (adipokines, protein, lipid, and miRNA) also occur in response to WAT cellular stress during obesity as part of the hypertrophic adipocyte SASP [160,161,199,200]. In lean VAT, high- and middle-molecular-weight adiponectin is expressed on the surface of small EVs and greatly contributes to the stability of circulating plasma adiponectin levels. In exosomes derived from obese VAT in *ob*/*ob* mice or HFD-fed C57BL/6 male mice, adiponectin levels were drastically reduced (up to 40-fold) compared to lean WT or standard chow-fed control mice [83] (Figure 9E,F). Adiponectin was the only adipokine carried by VAT-derived exosomes from lean mice, with no leptin, IL-6, or TNF-α detected. Lean VAT-derived exosomes containing adiponectin, when administered intraperitoneally to HFD-fed mice, were able to achieve the following:

- Restore insulin sensitivity in the liver and skeletal muscle via binding to AdipoR1 and
- AdipoR2 and normalize insulin phosphorylation of AKT;
- Prevent hepatic steatosis, markers of hepatic injury (aspartate aminotransferase (AST)) and ectopic skeletal muscle fat deposition;
- Inhibit macrophage infiltration of liver and WAT and formation of macrophage Mac2^+^ crown-like structures in SAT and VAT;
- Decrease expression of MCP-1, TNF-α. IL-1β, and IL-6 in VAT;
- Decrease VAT and SAT fat deposition;
- Reduce adipocyte size to significantly smaller diameters in both VAT and SAT;
- Decrease hepatic gluconeogenesis by downregulation of the mRNA levels of hepatic
- phosphoenolpyruvate carboxykinase (PEPCK);
- Decrease hepatic PPARγ mRNA expression;
- Prevent HFD-induced weight gain [83].

VAT exosomes derived from adiponectin KO mice did not have these effects, and obese VAT-derived exosomes failed to restore palmitate-treated human hepatoma (HepG2) cell insulin sensitivity [83]. 

High-fat diet-induced obesity in C57B6J mice profoundly changed the circulating plasma exosome profile, with exosomal miR-122, miR-192, miR-27a-3p, and miR-27b-3p directly involved in the early stages of metabolic syndrome development [160]. This included glucose intolerance, insulin resistance, dyslipidemia, visceral fat accumulation, impaired FAO, and peripheral delivery of FFAs [160]. Elevated miR-223 was also found, which modulates macrophage phenotype and activation state [161]. In palmitate-induced adipocyte hypertrophy (3T3-L1 adipocyte model), exosomes containing miR-802-5p downregulated HSP-60, which resulted in oxidative stress and insulin resistance [195]. Hypoxic conditions in 3T3-L1 adipocytes increased exosomal proteins promoting lipid synthesis (ACC, G6PD, FAS). This was a 3–4-fold increase over normoxic conditions [195]. Increased ceramide induces the budding-in process and forms sphingomyelin. This is metabolized by sphingomyelinase into sphingosine and sphingosine-1-phosphate, promoting the formation of ILVs and biosynthesis of exosomes [201,202,203].

Cellular senescence is involved in adipose tissue inflammation, immune response, and impaired homeostasis. The accumulation of senescent cells also contributes to aging, obesity, atherosclerosis, hyperinsulinemia, NASH, and T2DM [53,204]. This is due to the hypertrophic adipocyte SASP spreading via exosomes and cytokines to other cells, including adipose tissue infiltrating macrophages, ADSC, cardiac and skeletal myocytes, pancreatic β cells, renal podocytes, vascular endothelial cells, hypothalamic neurons, and hepatocytes [21,52,56,57,59] (Figure 5 and Figure 8).

### 7.6. Obesity, Exosomes, and Macrophage Polarization

Macrophages constitute around 40–50% of cells in the lipid depot in obesity, compared to 5–10% in lean individuals [205]. There is both heterogeneity and plasticity in the ATMφ phenotype, which is determined by multiple stimuli in the WAT and vascular microenvironment. These include nutrients (glucose, palmitate, PUFA), pathogen-associated molecular patterns (PAMPs), membrane receptor TLR-4 ligands (DAMPs), insulin, cytokines (TNF-α, IFN-γ), adipokines, hypoxia, pH, ROS, exosomal cargo, miRNA (miR-27a, miR-34a, miR-155), and the adipocyte SASP secretome. Lean WAT and BAT in metabolically healthy individuals typically contain a predominant resident embryonic-derived macrophage phenotype (F4/80^+^, CD11b^+^, TIM4^+^, CD11c^−^, expressing PDGF-CC) or BMD M2-like macrophages (F4/80^+^, CD11b^+^, CD301^+^, CD206^+^), which are associated with the AT vasculature [206]. Crosstalk between perivascular mesenchymal cells and adipose tissue macrophages modulates WAT inflammation and macrophage migration and accumulation in obesity [206]. 

M2-like macrophages utilize FFAs from lysosomal lipolysis, FAO, and mitochondrial biogenesis. The M2 phenotype is promoted by SIRT-1; adiponectin; Group 2 innate lymphoid cells (ILC2); anti-inflammatory Th2 cytokine (IL-4, IL-13) release from CD4^+^ T-helper type 2 (Th2) cells and adipocytes; and the transcription factors Krüppel-like factor 4 (KLF4), STAT6, PPARδ, PPARγ, and the PPARγ transcription coactivator-1β (PGC-1β). M2-like polarized macrophages help to maintain adaptive thermogenesis, UCP-1 expression, and beige adipogenesis in WAT and also secrete anti-inflammatory cytokines (transforming growth factor beta (TGFβ), IL-10, IL-1 decoy receptor) [207]. Inhibition of FAO and oxidative phosphorylation in macrophages by orlistat or miR-34a prevents M2 polarization and inhibits M2 arginase activity. Arginase in M2 macrophages degrades L-arginine into urea and ornithine and prevents inducible nitric oxide synthase (iNOS) production of NO from L-arginine and oxygen. Inducible NOS is a characteristic of inflammatory M1 macrophages [208]. PPARγ decreases the expression of C-C chemokine receptor type 2 (CCR2) on monocytes, which is the receptor for monocyte chemoattractant protein1 (MCP-1/CCL2), thereby minimizing bone marrow-derived monocyte (BMDM) chemotaxis and infiltration of WAT [209,210,211]. 

### 7.7. Macrophage Phenotypes

Increased chemotaxis signaling by senescent or dying hypertrophic adipocytes in obesity is associated with the activation and upregulation of circulating BMDM and their infiltration into WAT [211]. Here, they can be polarized into the following:

- M1 phenotype (F4/80^+^, CD11b^+^, CD11c^+^);
- Hybrid M1/M2 or CD9^+^ type (CD11b^+^, Ly6c^−^, and CD9^+^, found within CLS);
- Lipid-associated macrophages (LAMs, CD9^+^, CD63^+^, Trem2^+^), which are activated by exposure to glucose, palmitate, and insulin and are also known as metabolically activated macrophages (MMes) [211].

Obesity is also associated with increased sympathetic neuron-associated macrophages (SAM), which contain noradrenaline transporter Slc6a2 and noradrenaline degradation enzyme monoamine oxidase. SAMs blunt the effect of sympathetic neuron release of noradrenaline, which normally activates adipocyte β2-AR, adipocyte lipolysis, and FAO/thermogenesis [206,212,213]. Macrophage polarization changes from a 4:1 M2-to-M1 ratio in lean animals to a 1.2:1 ratio in obese animals during diet-induced obesity, as M1 macrophages are recruited into WAT [214].

M2 macrophages can be further divided into M2a–d subpopulations based on environmental stimuli, cell surface markers, and cell functions (Table 2) [211].

M1 macrophage polarization is classically triggered by endotoxemia (LPS), Th1 pro-inflammatory cytokines (TNF-α or IFN-γ), or by granulocyte–macrophage colony-stimulating factor (GM-CSF) [163,215]. However, MMes show lower inflammatory responses to LPS and IFN-γ than classical M1 macrophages and retain features of lipid-handling genes under the influence of PPARγ and LXR. These include ATP-binding cassette subfamily A member 1 (ABCA1) for reverse cholesterol efflux from MMes into apolipoprotein A1 and HDL, CD36 scavenger receptor for lipid uptake, and/or perilipin 2 [163,215]. Classically polarized M1 macrophages lose the capacity for lipid handling via the JAK/STAT signaling pathway, with the loss of ABCA1 in VAT. ABCA1 mRNA expression had a significant negative correlation with HOMA-IR (r = −0.44, *p* = 0.0003) and adipose tissue insulin resistance index (adipo-IR, r = −0.35, *p* =0.005) in patients with obesity (mean BMI = 44.5 kg/m^2^) [216]. Loss of ABCA1 expression is associated with intracellular cholesterol accumulation, TLR-4, and NLRP3 inflammasome activation in murine models and in patients with genetically inherited macrophage ABCA1 deficiency. Conversely, the expression of ABCA1 in VAT positively correlated with VAT adiponectin expression [217]. During obesity, the expression of ABCA1 can be inhibited in human macrophages and ABCA1/ABCG1 in murine macrophages by miR-23a-5p and miR-33a-5p, which leads to impaired reverse cholesterol transport into HDL, accelerated atherosclerosis, formation of foam cells and NAFLD [164,170].

M1 macrophages impair WAT adaptive thermogenesis by the inhibition of UCP-1 expression. This is achieved by direct adhesion of M1 macrophage α4-integrins to VCAM-1 on beige adipocytes, M1 release of TNF-α and IL-1β, and activation of JNK-1, IκB kinase-ε (IKKε), and TLR-4 signaling [207]. 

LAMs are important for lipolysis buffering in WAT, as they store and process lipids and prevent rapid release of NEFA into circulation [211]. However, macrophage lipid overload in WAT can be associated with lysosomal dysfunction and failure of lipophagy, leading to ER stress, mitochondrial dysfunction, and cellular senescence [208]. This is more prevalent in obese human VAT than SAT, with a significantly higher number of cells in VAT having a senescent phenotype, 70% of which were CD68-expressing macrophages. The number of SA-β-gal positive cells in VAT was positively correlated with BMI, insulin, and HOMA-IR [218].

Loss of SIRT-1 expression by senescent adipocytes results in impaired adipocyte adiponectin and IL-4 secretion and increased MCP-1 release, leading to M1-like polarization [63]. M1-like macrophage phenotypes have a transformed metabolism, utilizing the Warburg effect with upregulated pyruvate kinase muscle isoenzyme 2 (PKM2), aerobic glycolysis and lactate fermentation, NADPH synthesis via the PPP, and inhibition of apoptosis by PKM2 phosphorylating Bcl-2 [209,210,211]. M1 macrophages are characterized by a ‘broken’ Krebs cycle involving impaired isocitrate dehydrogenase (IDH) and succinate dehydrogenase activity. IDH normally catalyzes the oxidative decarboxylation of isocitrate to α-ketoglutarate and reduces NADP^+^ to NADPH, maintaining mitochondrial redox balance. Loss of IDH results in the accumulation of citrate, which is then used for fatty acid biosynthesis, including prostaglandins and itaconic acid. Itaconic acid increases cytosolic NADPH synthesis in the PPP and the antimicrobial properties of NOX and inhibits succinate dehydrogenase. Impaired succinate dehydrogenase activity in the Krebs cycle in M1 macrophages promotes succinate build-up, HIF-1α stabilization, and IL-1β expression. The arginosuccinate shunt enables NO synthesis from L-arginine, NADPH, and oxygen by iNOS. NO stabilizes HIF-1α and also inhibits the mitochondrial electron transport chain at complex IV, I, and III, with the generation of mitochondrial ROS (mROS), including superoxide and hydrogen peroxide. Generation of mROS and NOX-mediated cytoplasmic ROS is required for the respiratory burst and cytokine release (TNF-α, IL1-β, IL-6, IL-12, IL-23) during M1 phagocytosis. M1 macrophages do not require insulin for glucose uptake, and the release of ROS and IL-1β contributes to insulin resistance in insulin target cells, including adipocytes, hepatocytes, and myocytes [208,219]. 

Adipocytes exposed to high levels of saturated FFAs show increased activity of caspase 3 and Rho-associated kinase activities [220]. This increases the release of adipocyte-derived EVs, which promotes BMDM infiltration into WAT [220], including EVs containing MCP-1 mRNA [221]. In a mouse model, exosome-like vesicles released from AT facilitated communication between AT and macrophages, leading to the differentiation of circulating BMDM into pro-inflammatory M1-like macrophages. These M1 ATMφs subsequently secreted TNF-α and IL-6, contributing to insulin resistance [222]. In addition, exosomes derived from ATMφs in obese mice induced pancreatic β-cell insulin secretion and insulin resistance when injected into lean mice [208]. 

### 7.8. Exophagy

Adipocytes from obese mice showed *ATGL-independent* release of intact exosomal TAG with a doubled rate compared to lean mice (0.1% vs. 0.04% total adipocyte TAG/hour) [223]. Although this represented a small proportion of overall TAG turnover, this non-hydrolyzed TAG excreted in exosomes (exophagy) promoted differentiation of BMDM into an ATMφ-like phenotype with neutral lipid accumulation, multinuclear formation, lysosome biogenesis, and lipid catabolism [223]. Oxidized LDL promotes the formation of CD36/Na/K-ATPase signaling complexes in adipocytes, which increase exosomal secretion, leading to pro-atherogenic M1 phenotypes [224]. 

### 7.9. Exosomal miRNA and Macrophage Polarization 

In 2010, Ogawa et al. captured EVs containing adipocyte-specific mRNA gene transcripts for adiponectin, resistin, and PPARγ2 circulating in serum from normal-weight rats and showed miRNA and adipokine mRNA could be transferred in vitro from 3T3-L1 adipocytes into macrophages [225]. It was later shown that lean and obese WAT-derived exosomes have very different mRNA and miRNA profiles [78,109,137,169]. Adipocytes in HFD-fed mice and in genetically obese (*ob*/*ob*) mice showed evidence of senescence (increased p53 and *CDKN1A*) and increased release of the p53 transcriptional target miR-34a [137]. Elevated glucose and insulin in obesity increase the release of adipocyte-derived exosomes carrying Sonic Hedgehog and miR-34a, both of which promote M1 polarization [137]. Upregulation of miR-155 in adipocyte-derived microvesicles is also observed in obese mice. MicroRNA-155 inhibits the suppressor of cytokine signaling-1 (SOCS1), which results in an M1 macrophage phenotype by activation of STAT1 (transcription factor of IFN-γ) and suppression of STAT6 (transcription factor of IL-4) [169]. Adipocyte-derived exosomal miR-34a is found in much higher levels in obese VAT than in obese SAT in both humans and mice [137]. MicroRNA-34a inhibits M2 macrophage polarization by suppression of IL-4 and its downstream target KLF4 [137]. As a result of the miR-34a transfer, polarization of highly glycolytic M1-like pro-inflammatory macrophages was observed in obesity-induced WAT inflammation with associated collagen production and fibrosis. This was also thought to contribute to impaired WAT storage flexibility and increased ectopic fat deposition due to lipotoxicity [137]. Exosomal miRNA-34a suppresses the activity of FGF19/FGF21 by inhibiting the expression of β-klotho, which is a required co-receptor for both hormones and their promotion of BAT activity and WAT browning. MicroRNA-34a also reduces NAD^+^ levels, resulting in the suppression of SIRT-1 activity. Knockout of miR-34a in HFD-fed obese mice attenuated VAT fibrosis, local (WAT CLS formation/M1 polarization) and systemic (serum cytokines: TNF-α, IL-6, IL-1β, and MCP-1) metaflammation, insulin resistance, and hepatic steatosis. This resulted in a reversal of the diminished levels of circulating adiponectin and a 69% increase in WAT M2 polarization [137] (Figure 5).

### 7.10. WAT Exosomes, CLS, and Macrophages

Exosomes from obese VAT, more so than obese SAT exosomes, induce M1-like macrophage polarization, TNF-α, and IL-6 secretion and subsequent macrophage foam cell formation [226]. This is supported by research showing greater numbers of ATMφs, foam cells, and crown-like structures (CLS) in VAT compared to SAT in humans with obesity as well as genetically obese mice [227,228]. Apart from obesity-induced inflammation, ATMφs also participate in the mobilization of lipids from hypertrophic adipocytes by lysosomal acid hydrolase activities. Lipid droplet accumulation in ATMφs activates lysosome biogenesis without inflammation phenotype. This is associated with metabolic complications, including insulin resistance and hepatic steatosis [229], and is further promoted by lipid-laden EVs derived from adipocytes (exophagy) [223,230].

**Figure 10:**
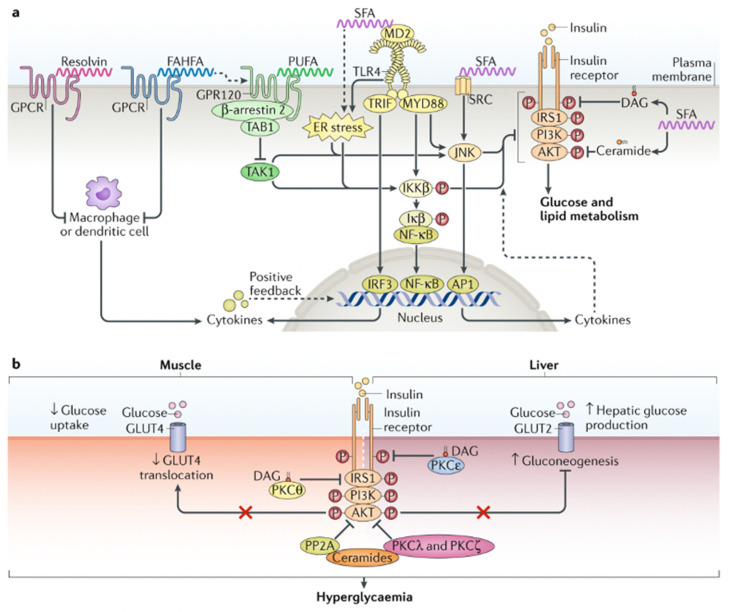
Lipids as signaling molecules that regulate metabolism. Several signaling lipids, including fatty acids, fatty acid esters of hydroxy fatty acids (FAHFAs), diacylglycerol (DAG), and ceramides, regulate insulin sensitivity. (**a**) Saturated fatty acids (SFAs) activate Toll-like receptor 4 (TLR4), possibly via its co-receptor, myeloid differentiation protein 2 (MD2), which, through the adaptor proteins TIR domain-containing adaptor-inducing interferon-β (TRIF) and myeloid differentiation primary response protein MYD88, increases the activity of pro-inflammatory transcription factors. TRIF activation promotes the nuclear translocation of interferon regulatory factor 3 (IRF3) to increase the expression of cytokines. MYD88 activation increases phosphorylation of inhibitor of nuclear factor-κB (NF-κB) kinase (IKKβ), which further phosphorylates inhibitor of NF-κB (IκB), leading to nuclear translocation of NF-κB to increase pro-inflammatory cytokine expression. MYD88 also activates Jun N-terminal kinase (JNK) to increase the activity of transcription factor activator protein 1 (AP1), thereby altering the expression of cytokines. Proinflammatory cytokines, through their receptors, further activate these proinflammatory transcription factors to establish a positive feedback loop for sustained inflammation. SFA-activated TLR4 and cytokine production impair insulin signaling through IKKβ and JNK activation. SFA-mediated activation of endoplasmic reticulum (ER) stress, the SRC–JNK pathway, and the incorporation of SFAs into DAG and ceramides also impair insulin signaling by inhibiting phosphorylation of the insulin receptor, insulin receptor substrate 1 (IRS1) or AKT. Polyunsaturated fatty acids (PUFAs) exert anti-inflammatory effects by activating G-protein-coupled receptor 120 (GPR120), which recruits β-arrestin 2 and sequesters TAK1-binding protein 1 (TAB1) to inhibit the TAK1-mediated activation of JNK and IKKβ. PUFA, FAHFAs, and resolvins may exert anti-inflammatory effects by activating G-protein-coupled receptors (GPCRs) in antigen-presenting cells such as macrophages to inhibit cytokine production. (**b**) DAG and ceramide may induce insulin resistance. DAG accumulates ectopically in insulin-resistant muscle and liver. In muscle, DAG-activated protein kinase Cθ (PKCθ) promotes the phosphorylation of IRS1 on Ser1101 in mice, impairing IRS1 phosphorylation on tyrosine and attenuating insulin signaling. In the liver, DAG-activated PKCε promotes phosphorylation of IR on Thr1160 to suppress insulin signaling. Elevated DAG impairs glucose uptake via reduced insulin-responsive glucose transporter 4 (GLUT4) in muscle, and glucose output via GLUT2 from the liver is increased; these changes induce hyperglycemia. Ceramides contribute to hyperglycemia by activating protein phosphatase 2 A (PP2A), which dephosphorylates AKT, stimulating PKCλ and PKCζ, which prevent AKT membrane association, thereby inhibiting AKT activity. Reproduced from Yang et al. (2018) [108].

Increased numbers of multinucleated giant cells (MGCs) and CLS are observed in obesity. More than 90% of WAT macrophages are localized around dead or senescent adipocytes [231]. These macrophages engulf lipid droplets and undergo fusion and formation of syncytia (MGC), which ultimately generates CLS [231]. Exposure to saturated FFAs, free cholesterol, oxidized phospholipids, and eicosanoids from ω-6 PUFAs increases ATMφ inflammation via ER stress, c-Src, and TLR-4 activation. Conversely, resolvins from ω-3 PUFAs are anti-inflammatory via GPCR signaling [108,213] (Figure 10).

The fusion of ATMφs to form MGCs is promoted in lipid-rich environments, as long-chain FFAs (e.g., palmitate) have a fusogenic effect when binding to macrophage CD36 receptors [232,233]. WAT MGCs or *adipoclasts* attempt to engulf large lipid remnants that cannot be cleared by single ATMφs [234] and are implicated in the clearance of stressed adipocytes during obesity, as well as providing efferocytosis of dead adipocytes [235]. However, oxLDL and cholesterol crystallization, rupture of phagolysosomal membranes, leakage of lysosomal enzymes, secretion of pro-inflammatory cytokines (IL-1β), and impaired lipid handling results in *frustrated phagocytosis*. This furthers NLRP3 inflammasome activation, ATMφ SASP, dyslipidemia, NASH, atherosclerosis, and metabolic syndrome [124]. The progressive loss of phagocytic capacity and increased SASP was found in senescent CD9^+^ macrophages in CLS, which share some features with foam cells (lipid droplet-laden macrophages) from atheromatous plaques [236,237]. 

## 8. Insulin Resistance, Senescence and Obesity: Mechanisms and Hypotheses

A high-fat/high-sugar diet and sedentary lifestyle promote the storage of excess energy in WAT (the *energy balance hypothesis* of obesity) [127]. This leads to pathological WAT hypertrophy, metaflammation, and lipotoxicity, with progressive insulin resistance and elevated HOMA-IR [127]. A high-saturated-fat diet also results in palmitate activation of hypothalamic TLR-4 receptors, leading to pro-inflammatory TNF-α, IL-1β, IL-6, and pIκBa signaling; hypothalamic inflammation, ER stress, and microglial senescence; central leptin and insulin resistance; and inappropriate appetite stimulation [238,239].

In some patients, high-glycemic carbohydrate diet-induced hyperinsulinemia and dyslipidemia may *precede* the development of obesity and insulin resistance and thus be a causative factor in, rather than just a consequence of, insulin resistance and lipogenesis (the *carbohydrate-insulin model*) [240]. In the presence of hyperinsulinemia and impaired skeletal muscle utilization of glucose, a paradoxical state develops of impaired hepatic glycogenolysis but abnormal gluconeogenesis and VAT FFA release. This may also be due to elevated circulating fructose derived from excessive exogenous sucrose or high-fructose corn syrup consumption in dietary food and beverages or from endogenous fructose synthesis (polyol pathway). The polyol–fructose–uric acid (PFU) pathway involves the conversion of excessive glucose to sorbitol by NADPH-dependent aldose reductase and then to fructose by NAD^+^-dependent sorbitol dehydrogenase with an associated release of uric acid and a fall in mitochondrial ATP. Importantly, aldose reductase is stimulated by dietary salt, alcohol, umami foods, or excessive simple carbohydrates and environmental factors, including ischemia, hypoxia, heat stress, uric acid, hyperosmolarity, or dehydration. High levels of fructose stimulate hepatic ChREBP, gluconeogenesis, and de novo lipogenesis, a process *independent* of insulin signaling [241]. Excessive fructose may thus contribute to visceral TAG deposition, central leptin resistance, impaired satiety, and abnormal foraging behavior, including a preference for high-fat foods. This comprises the *fructose survival pathway,* which is usually activated during environmental stress or in preparation for migration or hibernation. The *fructose survival hypothesis* has been proposed to unify the various obesity hypotheses, which also include the *seed oil hypothesis* and the *protein leverage hypothesis*, reviewed by Johnson et al. (2023) [240]. 

In addition, the excessive uric acid generated by the PFU pathway may also contribute to oxidative and ER stress by the production of hydrogen peroxide or superoxide during the conversion of xanthine to uric acid by xanthine oxidoreductase; activation of the renin–angiotensin–aldosterone systems, translocation of NOX-4 to mitochondria, and increased superoxide production; acetylation of carnitine palmitoyltransferase 1A (CPT1A), inhibition of mitochondrial aconitase, accumulation of citrate and stimulation of ATP citrate lyase and lipogenesis, formation of malonyl CoA, and inhibition of CPT1A/FAO; and depletion of NAD^+^, decreased SIRT1/AMPK activity, and promotion of cellular senescence [242,243]. Tissues that lack sorbitol dehydrogenase, including the kidneys, retina, and Schwann cells, are exposed to toxic levels of intracellular sorbitol. Activation of the PFU pathway due to prolonged hyperglycemia in T2DM, therefore, contributes to diabetic nephropathy, retinopathy, and peripheral neuropathy. Uric acid also promotes a positive feedback loop in the PFU pathway by directly promoting fructose metabolism by fructokinase and fructose synthesis by aldose reductase activity [242,243]. 

**Figure 11:**
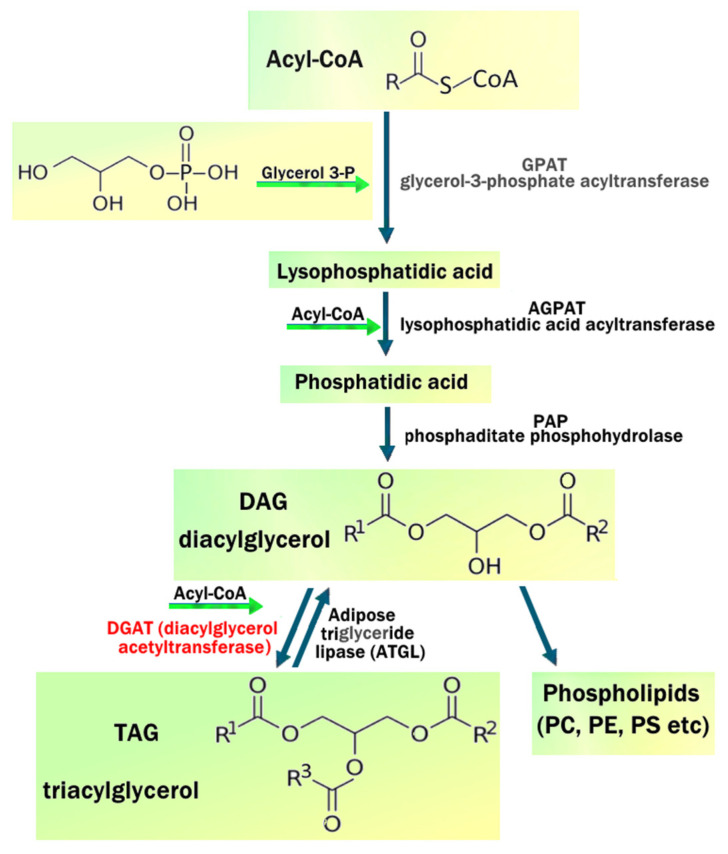
Schematic representation of glycerolipid metabolism in the synthesis of diacylglycerol (DAG) and triacylglycerol (TAG) from glycerol and acyl-CoA [246]. PC, phosphatidylcholine; PE, phosphatidylethanolamine; PS, phosphatidylserine.

Obesity and insulin resistance increase the rate of basal lipolysis (but not catecholamine-stimulated lipolysis) and release of NEFA from hypertrophic adipocytes [244]. WAT hypoxia leads to HIF-1α and HIF-2-dependent downregulation of PPARγ coactivator-1α (PGC-1α) and CPT1A, as well as HIF-1α mediated downregulation of medium- and long-chain acyl-CoA dehydrogenases (MCAD and LCAD). As a result, shuttling of FAs into the mitochondria and their β-oxidation is inhibited during hypoxia [245]. Increased cytoplasmic FFA, together with impaired mitochondrial FAO, results in the accumulation of LCFA-CoA, which is metabolized to DAG and ceramides [103,246] (Figure 5 and Figure 11).

Elevated intracellular ROS, saturated fatty acids (palmitate), fatty acyl-CoA, and DAG levels activate protein kinase C (PKC)-theta, IκB kinase β (IKKβ), and JNK, leading to IRS-1 Ser(307) phosphorylation [247,248,249,250,251,252,253]. IRS serine phosphorylation inhibits IRS/insulin receptor signaling and increases IRS degradation [248,249,250,251] (Figure 10). Intracellular DAG (a second messenger molecule) is also increased by angiotensin II agonizing the Gq-coupled type 1 angiotensin II receptor (AT1R), which activates NOX-4 and inhibits normal insulin-induced glucose uptake (by 80%) in adipocytes. This effect of the local WAT renin–angiotensin system (RAS) is reversed by AT1R blockade with losartan [100], c-Src inhibition, or N-acetyl cysteine [109]. 

### 8.1. VAT, Angiotensin II, Insulin Resistance, and Senescence

VAT has greater expression of renin–angiotensin system components than SAT, and angiotensin II secretion is enhanced by higher BMI, TNF-α, and insulin [252,254]. Angiotensin II impairs adipogenesis in VAT via PPARγ inhibition and promotes insulin resistance through activation of ERK1/2, which inhibits insulin phosphorylation of Akt. Angiotensin II causes vasoconstriction of WAT vasculature, resulting in further WAT hypoxia and hypoxia-related inflammation. Angiotensin II also increases adipocyte mitochondrial ROS, ER stress, and NF-κB release and promotes adipocyte senescence with associated SA-β-gal expression and release of exosomes. Treatment with an AT1R blocker (irbesartan, losartan, telmisartan) decreases WAT release of inflammatory cytokines (leptin, IL-6, and IL-17) and miRNA associated with ER stress (miR-708-5p, miR-143-3p) and improves adipogenesis (partial PPARγ agonist effect), insulin resistance, markers of adipocyte senescence, mitochondrial ROS, and secretion of anti-inflammatory adipokines such as adiponectin [252,254]. 

### 8.2. Hyperinsulinemia and Oxidative Stress

During hyperinsulinemia, aberrant phosphorylation of Ras-related C3 botulinum toxin substrate 1 (Rac1) by PI3K instead of its canonical target phosphatidylinositol 4,5-bisphosphate (PIP2) also leads to NOX-4 activation and ROS release in adipocytes [253]. Oxidative stress results in retromer signaling to the trans-Golgi apparatus to transport GLUT4 to lysosomes for their degradation instead of their translocation to the plasma membrane [253]. In skeletal muscle, normal activation of phosphatidylinositol 3-kinase (PI3K) is also required for insulin-induced GLUT4 translocation and subsequent transmembrane glucose intake [253]. PI3K regulates sterol regulatory element-binding proteins (SREBP), which promote lipid synthesis. Decreased IRS activity downregulates PI3K activity and, therefore, leads to insulin resistance and impaired utilization of glucose by skeletal muscle [102]. 

### 8.3. Loss of Adiponectin, Increased Ceramide and Insulin Resistance

During obesity, chronic inflammation, pre-existing insulin resistance, reduced AdipoR-dependent ceramidase activity, and altered gut microbiota/HFD contribute to elevated serum ceramide levels [54]. Ceramide promotes insulin resistance by inhibiting PI3K and anabolic Akt/PKB activity. This interferes with both insulin signaling and transmembrane glucose uptake, which induces plasma hyperglycemia and glucotoxicity. Ceramide also increases ER stress and apoptosis in pancreatic β cells in the presence of inflammatory cytokines and lipotoxicity, eventually leading to *decreased* insulin secretion, pancreatic endocrine failure, and late T2DM [108,246] (Figure 10).

### 8.4. Hepatic Insulin Resistance

Dysregulated WAT lipolysis also causes deposition of NEFA in the liver, contributing to non-alcoholic fatty liver disease (NAFLD) [255]. NAFLD further impairs insulin sensitivity and hepatic glycogen synthesis. Similar to skeletal muscle, accumulation of DAG in hepatic cells activates PKC, which decreases IRS2-associated PI3K-activity. This results in impaired insulin-induced hepatocyte glucose uptake and inhibits the ability of insulin to suppress hepatic gluconeogenesis. Decreased PI3K-activity also diminishes AKT2 activity, allowing forkhead box O (FOXO) to increase transcription of the rate-limiting enzymes of gluconeogenesis (phosphoenolpyruvate carboxykinase (PEPCK) and glucose-6-phosphatase (G6Pase)). Lipotoxicity thus results in elevated plasma glucose levels [103] (Figure 10).

### 8.5. Insulin Resistance and Inflammation

In obesity, poor oxygenation of hypertrophied WAT results in hypoxia and ER stress, particularly in CLS. ER stress induces UPR, WAT inflammation, and senescence. ER stress promotes insulin resistance through complex mechanisms, including a decrease in adiponectin levels, which increases mTOR activity, IRS phosphorylation, and leptin resistance [126]. Endoplasmic reticulum stress and mitochondrial dysfunction promote mitochondrial ROS and NOX-4 ROS formation, further activating the NLRP3 inflammasome. Other DAMPs involved in TLR-4/9 and canonical NLRP3 inflammasome activation in obesity include elevated palmitate and other saturated fatty acids, glucose, uric acid, C-reactive protein (CRP), ceramide, islet amyloid polypeptide, HMGB1, mitochondrial DNA, and cholesterol crystals/oxLDL [256] (Figure 9). Elevated oxLDL due to lipid peroxidation by ROS can stress adipocytes, causing the transformation of adipocytes to an insulin-resistant and inflammatory phenotype [257]. OxLDL also increases adipocyte exosome secretion, which further impairs metabolic function [224]. VAT metaflammation in obesity, as measured by mRNA levels of TNF-α, IL6, and MCP-1/CCL2, is associated with insulin resistance and cellular senescence. A composite score of circulating miRs 181b-5p, 1306-3p, and 3138 combined with HOMA-IR could reliably discriminate between patients with high and low levels of VAT metaflammation [258]. 

### 8.6. EVs and Insulin Resistance

Recent studies have demonstrated the relationship between EVs and insulin resistance [259]. Adipocyte-derived exosomal miR-27a has been reported to inhibit PPARγ expression in skeletal muscle, leading to insulin resistance [168] (Figure 5). By inhibiting preadipocyte differentiation into mature adipocytes via suppression of PPARγ, the ability to expand WAT lipid storage in obesity is limited, contributing to ectopic lipid deposition and lipotoxicity. In hepatocytes, TNF-α, IL-6, retinol-binding protein 4 (RBP-4), and macrophage migration inhibitory factor (MIF) secreted via AT-derived EVs inhibit insulin-induced Akt phosphorylation [259]. EVs released by hypoxic adipocytes can also inhibit 2-deoxyglucose uptake in other adipocytes, indicating the role of EVs in spreading insulin resistance among adipose tissues [260]. AT-derived EVs promote insulin resistance by downregulating IRS-1 and GLUT4 in adipocytes via miR-146b [167]. EVs from other cell types, including skeletal muscle cells, can also cause insulin resistance [261]. Adipocyte-derived EVs promote M1-like polarization, which increases IL-6 and TNF-a release through a TLR-4-dependent pathway [222]. Exosomes derived from F4/80^+^, CD11b^+^ M1 macrophages in VAT from HFD-fed obese C57BL6 mice carried miR-155, which inhibited PPARγ in muscle cells, hepatocytes, and VAT adipocytes [181]. This dysregulation of PPARγ and its downstream targets of GLUT-4 by exosomes contributed to in vivo and in vitro insulin resistance and glucose intolerance [181].

## 9. NAFLD, Hepatocyte Senescence, Exosomes, and NASH Progression

NAFLD is prevalent in obesity and is closely related to hepatocyte senescence [262]. The normal liver has between 3 and 7% senescent hepatocytes, but in end-stage NAFLD, this can increase to 50–100% [262]. The spectrum of NAFLD can include hepatic steatosis, NASH, liver fibrosis, or cirrhosis [262]. Steatosis occurs when hepatic lipid intake or synthesis exceeds the rate of lipolysis and lipophagy, leading to lipid droplet retention and hepatocyte “ballooning”. Exposure to saturated FFAs (palmitate) also inhibits the fusion of the lysosome and autophagosome, thereby preventing autophagy and promoting lipid droplet, p62, and ubiquitinated protein accumulation in hepatocytes [263,264,265]. 

In obesity, the release of FFA and cytokines (IL-6, IL-1β, PAI-1, TNF-α) from VAT into the portal vein may contribute to hepatic insulin resistance, hepatic triglyceride deposition, and NAFLD—*the portal hypothesis*. Other contributing factors include increased hepatic gluconeogenesis and de novo lipogenesis, impaired hepatic FAO, and dysfunctional synthesis of vLDL [266,267,268]. When hepatic glycogen stores are saturated in obesity, hepatic insulin resistance drives gluconeogenesis from multiple precursors, including lactate, pyruvate, glycerol, glutamine anaplerosis, and propionate. Excessive gluconeogenesis and uncontrolled fructose metabolism promote hepatocyte ER stress, mitochondrial ROS generation, UPR, inhibition of AMPK, activation of lipogenic transcription factors (SREBP-1c, ChREBP, and liver X-receptor (LXR)), AGE formation, mitochondrial citrate shuttling/palmitate synthesis, hepatic de novo lipogenesis, and NASH [253,269]. 

### 9.1. Lipotoxic Hepatocytes and EVs

Lipotoxic hepatocytes display features of SASP and have increased secretion of EVs [173]. Toxic lipids, including palmitate, stearic acid, and lysophosphatidylcholine (LPC), stimulate hepatocyte EV release, whilst non-lipotoxic FFAs (oleic acid) inhibit EV release [228]. Increased cholesterol accumulation in the liver also induces further hepatocyte ER stress, mitochondrial dysfunction, and ROS generation [270]. Release of EVs from lipotoxic hepatocytes mediates hepatic inflammation, angiogenesis, and fibrosis via ER stress sensor IRE1α, stress kinase MLK3, and TRAIL-R2 signaling cascade [228]. Elevated release of miR-122 and miR-192 has been observed in murine models of NASH [173]. In murine and human studies, circulating hepatocyte mitochondrial DNA derived from EVs activates TLR-9 via TNF-α and contributes to the development of NASH [21,271,272].

### 9.2. EVs and NASH

Vanin-1 containing hepatocyte EVs can mediate endothelial cell migration and neovascularization in NASH [172]. Macrophage infiltration and activation are also features of NASH. The formation of cholesterol crystals due to excessive hepatocyte oxLDL/cholesterol accumulation promotes Kupffer cell recruitment, activates the NLRP3 inflammasome via DAMPs receptors (TLR-2/4), and leads to the formation of foam cells and CLS. Hepatic CLS distinguishes simple steatosis from NASH on histology [273,274]. EVs from lipotoxic hepatocytes containing CXCL10 stimulate BMDM chemotaxis and infiltration to the liver [275]. Palmitate-induced EVs containing ceramide also activate macrophage chemotaxis [220]. In addition, macrophage activation is achieved by TRAIL contained in EVs [228]. MiR-192-5p in hepatocyte-derived EVs can stimulate M1-like macrophage activation, also contributing to the progression to NASH [171]. Apart from NASH progression due to exosomal upregulation of inflammation, exosomal miR-128-3P derived from lipotoxic hepatocytes inhibits PPARγ and directly mediates hepatic stellate cell profibrogenic activation. This promotes α-SMA, TGF-β, and CTGF release and progressive liver fibrosis and NASH cirrhosis [172] (Figure 5).

## 10. Adipocyte-Derived Exosomes and Hypothalamic Regulation of Energy and Appetite

Energy homeostasis, appetite, and body weight are controlled by anorexigenic pro-opiomelanocortin (POMC) and orexigenic neuropeptide Y/Agouti-Related Peptide (NPY/AGRP) neurons in the hypothalamic arcuate nucleus [180,185,195]. Activation of POMC neurons suppresses appetite, whilst mTOR signaling in POMC neurons reduces POMC expression and triggers hyperphagia-induced obesity. Because exosomes have a lipid bilayer membrane, they can cross the blood–brain barrier and be internalized in POMC neurons. Exosomes from visceral adipocytes in obese HFD mice contained the lncRNA metastasis-associated lung adenocarcinoma transcript-1 (MALAT1), which activated mTOR signaling through miR-181b and miR-144 in hypothalamic POMC neurons. These exosomes had the effect of increased appetite and weight gain when administered to lean mice. Conversely, administering VAT-derived exosomes from lean mice to obese mice resulted in weight loss and inhibition of food intake due to decreased hypothalamic mTOR expression and increased POMC activation [180,185,195]. This confirmed the importance of VAT-derived exosomes and the endocrine effects of SASP in obesity pathogenesis and weight recidivism [180,185,195]. 

## 11. Obesity, FAO, Thermogenesis, and Exosomes

Obesity is a state of dysfunctional TAG lipolysis, defective β-oxidation of fatty acids, and decreased cold- or diet-induced thermogenesis, with mechanisms including the following:

- Hypoxia-induced inhibition of adipocyte mitochondrial MCAD, LCAD, and CPT1 [276,277];
- VAT HIF-1α-induced suppression of SIRT2-NAD^+^ and PGC-1α leading to a decrease in oxidative lipid catabolism and mitochondrial biogenesis [278];
- Diminished adaptive thermogenesis, TAG lipolysis, and FAO in BAT and beige SAT due to inhibition of UCP-1 by polarized M1 macrophages during WAT metaflammation [207];
- Impaired thermogenic (brown/beige) fat function due to loss of UCP-1-independent futile substrate cycling/ATP consumption, including creatine/phosphocreatine cycling; sarcoplasmic/endoplasmic reticulum calcium ATPase (SERCA)/calcium pumping; and TAG lipolysis/fatty acid re-esterification cycling [112,207];
- Impaired FAO due to hyperglycemia, ATGL inhibition by hyperinsulinemia, and HSL inhibition by fatty acyl-CoA and oleic acid (*reverse Randle effect*) [279].
- Inadequate antioxidant, polyphenol, and fermentable fiber ingestion in inflammatory Western obesogenic diets [54]. Phytonutrients are required to combat postprandial oxidative stress; maintain insulin sensitivity and healthy gut microbiome; and modulate mitochondrial function, FAO, and lipid metabolism [280,281,282,283,284,285,286];
- Catecholamine-induced downregulation of adipocyte β3-adrenergic receptor mRNA and protein due to chronic activation of the sympathetic nervous system in obesity (*homologous desensitization*) [287,288];
- Impaired adipocyte response to catecholamine stimulation related to chronic TNF-α exposure and downregulation of adipocyte β3-adrenergic receptor mRNA (*heterologous desensitization*) and increased SAM (MAO) activity in WAT [105,206];
- Stimulation of activin B and growth differentiation factor 3 (GDF-3) receptor (ALK7) by nutrient overload, which results in downregulation of adipocyte β-adrenergic receptor expression and signaling (*catecholamine resistance*), impaired lipolysis and adipocyte hypertrophy [95];
- Decreased BAT mass, batokines, and UCP-1 activity in obesity, aging, and hyperglycemia [289,290,291];
- HFD-induced obesity in C57BL/6J mice stimulated the release of exosomal miR-27b-3p from VAT with an associated 7-fold decrease in UCP-1 and decreased white adipocyte browning and thermogenesis. This resulted in VAT adipocyte hypertrophy, visceral fat accumulation, and weight gain. The effects of miR-27b-3p could be inhibited by injection of lentiviruses encoding the antisense to miR-27b-3p (anti-miR-27b-3p [292].

## 12. Effects of Weight Loss on SASP, Exosomal miRNA and Insulin Resistance

Healthy WAT in lean individuals maintains insulin sensitivity, oxidative phosphorylation of glucose and fatty acids by skeletal and cardiac muscle, and normal hypothalamic regulation of energy balance and thermoregulation by the release of adiponectin, anti-inflammatory adipokines, and beneficial exosomal mRNA [293]. Adiponectin and SIRT-1 exist in a positive feedback loop and are suppressed in obesity due to ER stress, DNA damage, NAD^+^/NADH redox imbalance, and cellular senescence. Upregulation of adiponectin and SIRT-1 expression after sleeve gastrectomy in a murine model of sequential HFD-induced obesity and streptozocin-induced T2DM resulted in browning of SAT via PPARγ, PGC-1α, and UCP1 activation [293]. 

Successful weight loss in humans after Roux-en-Y gastric bypass bariatric surgery is associated with significant short-term improvements in SAT baseline gene mRNA levels involving inflammation (IL-6, TNF-α, MCP-1/CCL2), ER stress (eIF2α, calreticulin), adipogenesis (PPARγ), energy homeostasis (adiponectin and AMPK), cellular response to oxidative stress (SIRT1, SIRT3, and NFE2L2), mitochondrial biogenesis (PGC1α), and amino acid metabolism [general control nonderepressible 2 (GCN2)] [294]. Bariatric surgery significantly improved total body weight (by 27.5%), lymphocyte telomere length (by 12%), oxidative DNA damage (by 54%), lipid peroxidation (plasma MDA levels), and pro-inflammatory cytokines associated with SASP (CRP, serum amyloid A1 (SAA1)) in patients at 6 months after surgery [295]. 

**Figure 12:**
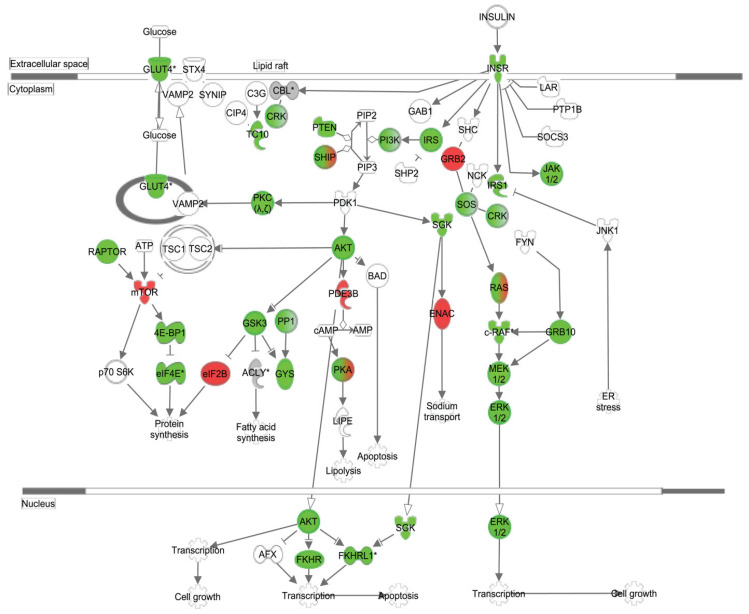
mRNA targets of microRNAs correlated to altered insulin resistance after surgery. Green color indicates predicted negative correlation of mRNA transcripts (i.e., positive correlation of microRNAs) to HOMA change following surgery, while red color indicates the reverse relationship (r > 0 for mRNA targets and r < 0 for microRNAs). Genes in grey and marked with an asterisk (*) represent targets of multiple microRNAs that have different correlational directions in relation to HOMA change. Reproduced from Hubal et al. (2017) [299].

Substantial differences in circulating miRNA are also found in responders and non-responders to bariatric surgical and dietary interventions in obesity [296]. This is related to the effect of exosomal miRNAs on the differential expression of their matching mRNAs, which control inflammation, fat metabolism, and adipogenesis in lean and obese phenotypes [297]. For example, acute weight loss from the energy restriction of very-low-calorie diets (<800 Kcal for 4 weeks) was able to normalize the exosomal profile of 7/13 dysregulated circulating miRNAs in women with obesity compared to lean individuals, including miR-34a, miR-208, miR-193, miR-320, miR-433, miR-568, and miR-181a [298]. Changes in exosomal miRNA controlling the insulin receptor signaling pathway and glucose transport in women one year after Roux-en-Y gastric bypass surgery correlated with successful weight loss (BMI, −18.6 ± 5.1 kg/m^2^; *p* < 0.001) and ameliorated insulin resistance (HOMA-IR: 1.94 ± 0.6 presurgery, 0.49 ± 0.1 postsurgery; *p* < 0.001) [299] (Figure 12).

However, after moderate weight loss (~10% TBW) with dietary and exercise interventions or even substantial weight loss (~22% TBW) after bariatric surgery, the cellular senescence/SASP/exosome profile of post-obese WAT in some patients may not return to that of lean WAT [300,301,302,303,304]. 

For example, impaired preoperative expression of local adiponectin (<5.1 µg/mg) in VAT, rather than circulating plasma or SAT adiponectin levels, predicted poorer magnitude of excess weight loss (EWL%) in women after bariatric surgery (98% accuracy, 100% sensitivity, 94% specificity, *p* = 0.010) [300]. This suggests that obesity causes permanent changes in the WAT secretome [301]. This is a risk factor for *weight cycling*, particularly if patients resume consumption of unhealthy Western-type diets or do not generate SIRT-1, AMPK, adiponectin, and beneficial myokine release through exercise. It is thought that exposure to palmitate after initial weight loss triggers innate metabolic ‘immune memory’ in previously sensitized adipose tissue macrophages. This leads to increased DNA methyltransferase activity and methylation of promoter CpG sites, TNF-α release, and resumption of metaflammation and weight regain [305]. Weight cycling is also related to the persistence of senescent programmed death receptor 1 (PD-1)^hi^ CD4^+^ T cells and CLS in VAT, which can actually *progress* during weight loss. This was shown in HFD-fed (60% kcal from fat) obese C57BL/6 mice when they were placed on a normal chow diet (10% kcal from fat) for 8 weeks in comparison to obese animals that continued HFD feeding [305]. This was despite final body weights and VAT mass in the normal chow-fed weight loss animals being similar to those of the lean control animals, as well as reversal of hepatic steatosis and VAT adipocyte hypertrophy. Senescent CD153^+^ PD-1^hi^ CD4^+^ T cells persisting in previously obese VAT produced large amounts of osteopontin as part of the SASP. This resulted in continued M1-like polarization of VAT macrophages with a Th1 response and insulin resistance [305,306]. 

Senescence-associated T cells in VAT from young adult HFD obese C57BL/6J male mice had a similar phenotype to splenic T cells from 80-week-old aged male mice, with increased p21 and γ-H2AX [307]. This suggested that the DDR contributed to senescence in both diet-induced obesity and aging. In contrast to shorter-term restriction, long-term caloric restriction (60% kcals of normal chow) for 28 weeks (~12 human years) in aged mice (52W) was found to reduce senescent VAT and splenic PD-1^+^ CD4^+^ and PD-1^+^ CD8^+^ T cells; improve glucose tolerance, insulin resistance, fasting serum insulin, and leptin levels; lower weight; and increase serum adiponectin levels, as compared to normal chow-fed aged mice. The M1 polarization of macrophages and formation of CLS was also reduced by long-term CR. Ablation of PD-1^+^ senescent cells in aged mice with PD-1^+^ cell-depleting antibodies had similar effects to long-term caloric restriction, with improved metabolic health, preservation of VAT M2 macrophages, and lower VAT M1 macrophage numbers [307]. 

Similarly, another murine study used CD153-CpG vaccination to successfully remove senescent CD153^+^ CD44^high^ CD62L^low^ PD-1^+^ CD4^+^ T cells in the VAT of HFD-induced obese young adult C57BL/6J mice, which improved insulin sensitivity, glucose tolerance, and HOMA-IR and decreased F4/80^+^ M1 macrophage and CD153^+^ senescent T-cell accumulation in VAT CLS [308]. PD-L1 gene transcription is enhanced by activation of cGAS-STING, NF-κB, the NLRP3 inflammasome, and IFN-γ/JAK/STAT pathways, which leads to impaired immunosurveillance and decreased clearance of senescent cells during aging and obesity [309]. Senescent cells also release exosomes coated with PD-L1 protein, which promotes senescence in surrounding cells in a paracrine fashion by PD-L1 binding to its PD-1 receptor on effector cells, including cytotoxic T cells and NK cells. Increased adipocyte leptin secretion in obesity promotes STAT3, CPT1B, and FAO in VAT CD8^+^ T cells with inhibition of glycolysis. Glycolysis is required for the effector functions of T cells and is inhibited by oxidation of the abundant SFAs in VAT. Inhibition of selective autophagy by mTOR activity also inhibits the degradation of PD-L1 protein, which further promotes T-cell senescence and impaired activity of cytotoxic CD8^+^ T and NK cells [309]. 

GLP-1RA treatment (s/c semaglutide) in Western diet-fed (chow containing 40% kcal from fat and 20% kcal from protein plus added sucrose) obese C57BL/6 mice resulted in substantial weight loss but did not improve hepatic steatosis or metabolic health in animals continued on a Western diet [310]. These animals also showed impaired CD8^+^ T-cell function and impaired response to immune checkpoint inhibitors (ICI). In comparison, Western diet-induced obese mice placed on a low-fat diet showed improved hepatic steatosis, serum cholesterol, CD8^+^ T-cell cytotoxicity, and response to ICI [311]. The problem of rapid weight regain and the return of metabolic syndrome after cessation of semaglutide obesity therapy if patients did not engage in healthy lifestyle interventions was highlighted in the extension study of the STEP 1 clinical RCT [310]. This may be related to persistent exposure to palmitate and impaired clearance of senescent cells, contributing to ongoing VAT inflammation and weight cycling [310]. 

Activin A is released from senescent adipocyte progenitors as part of the SASP cytokine component in both humans and mice and is elevated in aging, HFD-induced obesity, and weight cycling [204,312]. Activin A is a negative regulator of skeletal muscle mass by ligand activation of activin receptor type IIB (ActRIIB) and impairs adipogenesis by inhibiting *CEBPα* and *PPARγ* [95]. Other ligand agonists of ActRIIB include GDF8 and myostatin, and antagonists include follistatin (activin-binding protein) and bimagrumab (human monoclonal ActRII antibody). Removal of *p16^Ink4a^* senescent adipocyte progenitor cells by genetic ablation (INK-ATTAC) or JAK/STAT inhibition in murine models resulted in decreased activin A and improvements in insulin sensitivity, hemoglobin A1c (HbA1c), and adipogenesis [204,312]. 

A regain of weight after weight loss interventions (calorie restriction, incretin agonists) is associated with an increase in fat mass but not lean muscle mass. This can result in sarcopenic obesity and myosteatosis in weight cycling and can potentially be prevented by high-protein diets and a combination of aerobic exercise and resistance training [313,314,315]. The benefit of the preservation of skeletal muscle mass during weight loss due to calorie restriction and GLP-1R agonists was recently demonstrated in a bimagrumab/semaglutide trial in HFD-fed obese male C57Bl/6J mice. Combined treatment with bimagrumab and semaglutide resulted in a 70% loss of fat mass and no loss of lean mass. Semaglutide monotherapy resulted in a 50% loss of fat mass and a 10% loss of lean mass compared to control animals [316]. This is particularly relevant to future interventions in older patients with obesity who are already at risk of sarcopenic obesity and myosteatosis from lipotoxicity, weight cycling, and worsening of functional decline. In 2022, the European Association for the Study of Obesity (EASO) and the European Society for Clinical Nutrition and Metabolism (ESPEN) defined criteria for the definition of sarcopenic obesity, including measurement of skeletal muscle mass (appendicular lean mass/weight) and strength (hand grip strength, chair stand test) [317]. In population-representative data of North Americans over the age of 60 years, 28.3% had sarcopenic obesity. A proportion of patients may also be unsuitable for bariatric surgery due to age and obesity-related comorbidities and thus require tailored medical management integrated with exercise and nutrition programs [318,319]. 

## 13. Interventions in Obesity and SASP

1. Changing highly processed, energy-dense, inflammatory Western diets to fiber, ω-3 fatty acid- and phytonutrient-rich, anti-inflammatory Mediterranean-type diets [54,320,321,322]. This is important to both achieve and maintain established weight loss due to the persistence of senescent myeloid cells in WAT and the risk of weight cycling [305]. Treatment with marine ω-3 fatty acids eicosapentaenoic acid (EPA) and docosahexaenoic acid (DHA) increases exosomal adiponectin [323] and also improves M1 macrophage-induced SAT inflammation [323];
2. Time-restricted eating and intermittent fasting promote autophagy and clearance of senescent cells [324]. However, persistent VAT metaflammation and retention of senescent LAMs and T cells mediated by adipocyte p53 activation may predispose patients to weight cycling after weight loss from intermittent fasting and short-term caloric restriction without resistance exercise [323];
3. A combination of aerobic and resistance exercise, which increases thermogenesis, FAO capacity, skeletal muscle mass, adiponectin/SIRT-1, and beneficial exerkine (myomiRs, myokines, adipokines, batokines) and HSP release and decreases VAT volume and senescence [5,279,325,326,327]. Moderate exercise promotes hormesis, the development of adaptive responses to repeated sub-lethal cellular stress [13];
4. Weight loss, which decreases VAT volume, inflammatory adipokine, FFA, and exosome release and improves OSA, NAFLD, insulin resistance, and glycated hemoglobin (HbA1c) levels [5,325,328,329];
5. Senotherapeutic agents include senolytics, which induce apoptosis in senescent cells without affecting proliferating or quiescent cells, and senomorphics, which suppress the SASP [82,330,331,332]. Removal of only 30% of senescent cells by senolytic therapy was able to alleviate obesity-associated metabolic dysfunction and age-related functional decline in pre-clinical models [318]. Senolytics include Bcl-2 inhibitors (Navitoclax), the fruit and vegetable flavonoid antioxidants fisetin, quercetin, and luteolin, or intermittent dosing with combined dasatanib (Src family tyrosine kinase inhibitor) and quercetin (inducer of apoptosis). Dasatinib selectively targets senescent adipocyte progenitors, whereas quercetin targets senescent endothelial cells, preadipocytes, and mature adipocytes, promoting AMPK and SIRT-1 activity, which inhibits NF-κB, IL-1β, MCP1, and miR-155-5p signaling [333,334].

Fisetin is found in strawberries, apples, persimmons, grapes, onions, cucumbers, and herbs, and it has a chemical structure similar to quercetin. Fisetin has been shown to be effective at removing *p16^Ink4a^*-expressing senescent adipocytes, c-Kit^+^ stem/progenitor cells, CD4^+^ and CD8^+^ T cells, NK-1.1^+^ NK cells, and CD146^+^CD31^+^ endothelial cells but not macrophages in the WAT of aged C57BL/6 mice. Fisetin significantly reduced the percentage of SA-β-gal-positive cells and expression of the SASP factors IL-6, IL-8, and MCP-1 in cultures of human omental WAT explants. These were obtained during surgery in patients with a BMI > 25 kg/m^2^ [16]. Luteolin is found in celery, capsicums, lemons, pumpkin, lettuce, spinach, and herbs (sage, thyme). It effectively inhibits senescence by a mechanism different from fisetin by potently binding to CDK6 and inhibiting the effect of p16. Luteolin/*Salvia haenkei* extract was shown to reduce markers of senescence (p16, p27, γH2AX and 53BP1) in aged C57BL/6 mice and also prevent chemotherapy (doxorubicin)-induced cellular senescence and oxidative stress in young adult mice. Luteolin also increases glucolipid metabolism (↑PPARγ, GLUT4), BAT and beige WAT thermogenesis (↑PGC1α, UCP-1), FAO (↑p-ACC, p-AMPK, CPT-1), VAT lipolysis (↑AMPK, SIRT-1), autophagy, and apoptosis (↑Caspase 3/8, *↓*Bcl-2/Beclin1) and decreases WAT and liver lipogenesis (*↓*SREBP1, FASN, ACC), M1 and MMe macrophage polarization (↑p-STAT6,*↓*p-STAT3), NF-κB-mediated inflammation, and ROS (↑NFE2L2) in C57BL/6 mice models of obesity, NAFLD, and T2DM [335,336]. Navitoclax effectively inhibits Bcl-xL, Bcl-2, and Bcl-w but not Mcl-1. However, Navitoclax has the potential side effects of severe thrombocytopenia or neutropenia [333,334].

Senolytic therapy with dasatanib + quercetin (D + Q) in HFD-induced murine obesity is associated with improvement in cellular senescence markers, insulin sensitivity, metabolic syndrome, metaflammation, WAT macrophage infiltration, and adipogenesis in preclinical studies [204,337]. Age-related insulin resistance, fasting glucose, hypernefemia and senescence-associated β-galactosidase, expression of *p16* and *p21* gene and P16 protein, pro-inflammatory SASP genes (*mcp1*, *tnf-α*, *il-1α*, *il-1β*, *il-6*, *cxcl2*, and *cxcl10*), CLS, and abundance of T cells and macrophages in VAT were also significantly improved in aging male C57BL/6 mice by D + Q treatment [338]. However, sexual dimorphism in responses to long-term fisetin or D + Q senolytic therapy was recently shown in young adult C57BL/6 mice. Male mice had improvements in serum adiponectin, metaflammation (TNF-α, IL-6), glucose tolerance, cognitive function, VAT senescence, and SASP with fisetin, but female mice showed no response (fisetin) or adverse responses (D + Q) to senolytics. This was thought to be related to increased early metabolic aging in male mice; the protective effects of estrogen against senescence; VAT inflammation and insulin resistance; and different responses to lipid handling, SAT deposition, and BAT thermoregulation in female mice. A further proposed explanation for the lack of quercetin effect in younger or female mice was quercetin being more effective in the presence of iron or copper, which tends to accumulate in aged or senescent cells [11,306].

A small clinical study of D + Q in patients with diabetic renal disease showed a substantial decrease in WAT SA-β-gal^+^ and p16^INK4A+^ cells, WAT CLS, and CD68^+^ ATMφ numbers and an increase in adipocyte progenitor cells [332]. Senomorphic agents include metformin, resveratrol, or rapamycin, which inhibit mTOR by upstream promotion of SIRT1 and AMPK, thereby inhibiting SASP cytokine-induced metaflammation and activating autophagy. Metformin also reduces mitochondrial dysfunction by improving mitochondrial biogenesis (PCG-1α), mitochondrial dynamics (Drp1, MFF), and mitophagy (PINK1-Parkin pathway). However, proof of the long-term benefit and safety profile of senolytic or senomorphic treatments in human obesity in large clinical trials is required [31,82,330,331,332,339] (Figure 5).

6. AT1R blockade which improves adipogenesis, adipocyte senescence, SASP, metaflammation, and insulin resistance [252];
7. Long-acting GLP-1RAs, which improve obesity-induced lipotoxicity and glucotoxicity, alleviate related pancreatic β-cell and hypothalamic ER stress, and decrease peroxide-induced cellular senescence [340,341];
8. GLP-1RAs inhibit macrophage foam cell formation and atherosclerosis and angiotensin II activation of Rac-1/NOX production of superoxides and vascular smooth muscle senescence [342];
9. GLP-1RAs or caloric restriction improve NAFLD by inducing AMPK/mTOR-mediated autophagy and improving autophagy-dependent lipid degradation and hepatocyte senescence [343,344,345];
10. Use of the SGLT2 inhibitor canagliflozin in HFD-induced obese C57BL/6 mice reduced VAT senescence (attenuated SA-β-gal activity and *Cdkn1a* expression), oxidative stress, metaflammation (numbers of CLS), T-cell anergy and inhibition of clearance of PD-L1 expressing senescent cells, and metabolic dysfunction (glucose intolerance and insulin resistance). Correction of hyperglycemia by insulin therapy or a normal chow diet in HFD-fed obese control animals did not improve VAT senescence. In fact, insulin treatment *increased* VAT senescence and SASP markers, possibly via activation of Akt/mTOR pathways. The senolytic effect of canagliflozin was partially mediated by increased AMPK, which, like metformin, is a known inhibitor of mTOR. In humans with obesity and T2DM, a recent meta-analysis showed the combination of SGLT2 inhibitors and GLP-1RAs may be synergistic in improving insulin resistance, weight loss, and systolic blood pressure. Their combined effects on cellular senescence in humans remain to be shown [346,347];
11. Inhibition of exosome-mediated transmissible ER stress and SASP in adipocyte hypertrophic obesity by blocking ceramide-induced exosome biogenesis with sphingomyelinase inhibitors (GW4869) or glutathione [52,194];
12. Using exosomes to deliver beneficial miRNA/RNA mimetics, small interfering RNA (siRNA), or antisense anti-miRNA oligonucleotides (antagomiRs, ASOs) to treat obesity, metabolic syndrome, or atherosclerosis [160]. Exosomal delivery has the advantage of 1000-fold lower dosages than naked or adjuvant-bound nucleotides, together with potential target cell specificity and lower off-target side effects [160]. Exosomal surface proteins can also be engineered for increased tropism for specific recipient cell surface receptors. For example, the engineered fusion protein lysosome-associated membrane protein 2b (Lamp2b)/rabies virus glycoprotein peptide (RVG) improves exosomal delivery across the blood–brain barrier and selective binding of RVG to the nicotinic acetylcholine receptor on hypothalamic neurons. This enabled the delivery of a plasmid encoding an AMPKα1-dominant negative mutant (AMPKα1-DN), which enhanced UCP1 and BAT thermogenesis [348].

Exosomal delivery can obviate the problems of viral vector-based mRNA delivery systems (adeno-associated virus (AAV), lentivirus), such as immunogenicity, cytotoxicity, or poor biodistribution [349]. Apart from using exosomes from M2 macrophages or healthy adipose tissue-derived stem cells, exosomes can be biologically engineered by loading with antagomirs or mimetics. This may be achieved by passive incubation, electroporation, or donor cell manipulation with CRISPR-Cas 9 components or cytokine treatments [349]. 

For example, plasma exosomes from lean C57BL/6J male control mice transfected with miR-122 and miR-192 antagomiRs and miR-133b-3p mimics were intravenously injected into HFD obese mice, resulting in alleviation of glucose intolerance and improvement of hepatic steatosis [350]. The exosomes were designed to match the exosomal miRNA pattern in mice exercised with high-intensity interval training (HIIT), with decreased obesity-related miR-122 and miR-192 and increased miR-133-3p, a muscle-specific myomiR. It was found that HIIT exercise, but not engineered exosome treatment in HFD obese mice, was able to improve metabolic flexibility (respiratory exchange ratio), cardiorespiratory fitness, and maximal VO2 consumption (VO2max). The miR-122 is known to increase cholesterol and lipid synthesis by upregulating *Srebf1* and downregulating *Sirt1* in the liver, and miR-133 inhibits fatty acid synthesis by targeting *Acaca* or *Acsl4.* The engineered exosomes, therefore, inhibited hepatic de novo lipogenesis and SAT triglyceride synthesis, reduced circulating triglycerides, modulated cholesterol biosynthesis, and improved glucose intolerance and insulin sensitivity [350].

Another study generated exosomes from a human THP1 macrophage line polarized with IL-4 to an M2 phenotype, enriched with miR-21/99a/146b/378a, and depleted in miR-33 [351]. These were taken up by adipocytes, BMDM, and naïve THP1 macrophages in vitro and promoted M2 macrophage polarization (*Cd206*, *Cd163 Arg1*, *Chil3*, *Retnla*), lipophagy, oxidative phosphorylation, increased mitochondrial membrane potential ΔΨm, mitochondrial activity, and beigeing during adipocyte differentiation. Importantly, the increase in mitochondrial ΔΨm in BMDM was associated with decreased MOMP and increased mitochondrial ATP production. When injected intraperitoneally into 20 week old Western diet-induced obese wild-type C57BL/6 mice and LDL receptor-deficient *Apoe*^h/h^ *Ldlr*^−/−^ mice, THP1 Mφ-IL-4 exosomes suppressed systemic inflammation, VAT and hepatic inflammation (F4/80^+^ CLS formation), aortic inflammation (atherosclerosis), inflammatory markers of M1 macrophages (*Tnf*, *Il1b*, *Mcp1*, and *Nos2*), hepatocyte lipid accumulation, and hepatic steatosis and substantially reduced NF-κB activation, mitochondrial oxidative stress (superoxide production), fasting glucose, and insulin levels. There was increased lipid catabolism, lipophagy (*Ulk1*, *Pnpla2*, *Lipe*, *Map1lc3a*, *Map1lc3b*) and autophagy, VAT beigeing and thermogenesis, cholesterol mobilization and efflux (*Abca1*, *Apoe),* PPARγ-induced expression of glucose transporter GLUT4 (*Slc2a4*), uncoupling protein 1 expression (*ucp1*), adiponectin expression (*Adipoq*), and adiponectin/leptin ratios (2-fold rise). Obese WT mice treated with THP1 Mφ-IL-4-derived exosomes showed significantly lower plasma cholesterol and triglyceride levels than THP1 Mφ-IL-4 exosome-treated *Apoe*^h/h^ *Ldlr*^−/−^ mice. The study demonstrated the future potential for using exosomes enriched with beneficial miRNAs to treat human obesity, metabolic syndrome, NAFLD, and atherosclerosis [351].

## 14. Conclusions

This review has examined the relationship between diet-induced obesity and the development of adipocyte/ADSC senescence and the SASP. Healthy white adipose tissue in lean individuals is characterized by small adipocytes with minimal macrophage infiltration; the release of plasma, exosomal HMW adiponectin, and beneficial miRNA; and maintenance of normal energy homeostasis, mitochondrial activity, and insulin sensitivity. Storage of excessive nutrients and cellular stress in hypertrophic adipocytes leads to prolonged DNA damage, mitochondrial dysfunction, ROS generation, loss of adiponectin secretion, maladaptive UPR and ER stress, activation of inflammasome pathways, and recruitment and polarization of metabolically activated macrophages. This is not resolved by antioxidant systems, heat shock response pathways, or PARP DNA repair mechanisms, resulting in transmissible cellular senescence via autocrine, paracrine, and endocrine signaling. Adipose tissue senescence is closely linked to VAT expansion, HuR, and SIRT-1 inhibition and increased release of exosomes, harmful exosomal miRNAs and PD-L1, pro-inflammatory adipokines, and saturated FFAs. The resulting lipotoxicity, insulin resistance, and diminished fatty acid β oxidation leads to fat deposition in non-adipocyte reservoirs, with persistent and progressive obesity, metabolic syndrome, and premature aging. The accumulation of senescent cells and the failure of their immunoediting and replacement by healthy progenitors is central to hypertrophic obesity and associated functional decline. Targeted interventions to treat obesity, deliver beneficial exosomal cargo/miRNA, and reverse adipose tissue senescence, SCAPs, and SASP have many interrelated and synergistic benefits. 

## Figures and Tables

**Table 1:** Summary of exosomal or circulating miRNAs/ncRNA in obesity and obesity-related diseases.

Exosomal miRNA	Exosomal Source	Target Transcription Factors	Target Organ	Effect	Research Subject	Refs.
miR-125a	ADSC	Repress DLL4 expression	Adipose tissue	Promote angiogenesis in response to WAT hypertrophy and associated hypoxia	Primary HUVEC, mice	[158]
miR-21	ADSC	Increased expression of HIF-1α and VEGF	Adipose tissue	Promote angiogenesis in response to WAT hypertrophy and associated hypoxia	DU145 cells	[183]
miR-132	ADSC	Inhibit SMAD7	Adipose tissue	Promote angiogenesis in response to WAT hypertrophy and associated hypoxia	mice	[165]
miR-122 miR-192 miR-27b-3p	Adipose tissue	Inhibit PPARα	Adipose tissue	Induce glucose intolerance, insulin resistance, dyslipidemia, VAT accumulation, impaired FAO, and peripheral delivery of FFAs	mice	[160]
miR-27a/b	Adipose tissue	Inhibit PPARγ and C-EBP/α	ADSC	Inhibit adipogenesis; *↓* SAT storage capacity	mice	[164]
miR-27a-3p	Adipose tissue	Inhibit PPARγ, GLUT4, and IRS-1	Skeletal muscle	Induce glucose intolerance, insulin resistance, dyslipidemia, VAT accumulation, impaired FAO, peripheral delivery of FFAs, and lipotoxicity	mice, human	[160,168]
miR-223	Adipose tissue	TLR4/FBXW7 axis	SVF of human VAT	Modulate macrophage phenotype/activation state and response to stimuli via effects on TLR4/FBXW7 axis	human	[161]
miR-802-5p	Adipocyte	Downregulate HSP60, ↑ C/EBP/α expression; enhance oxidative stress and phosphorylation of JNK and IRS1	Cardiac myocyte	Induce cardiac insulin resistance	mice	[166]
miR-34a	Adipocyte	Repress expression of SIRT-1 and Krüppel-like factor 4	Macrophage ADSC and VSM	Promote M1 polarization Suppress M2 polarization Activation of p53 and senescence in WAT, MSC, and VSM	mice	[137]
miR-155	Adipocyte	Inhibit suppressor of cytokine signaling-1 (SOCS1); activate STAT1 and suppress STAT6	Macrophage	M1 macrophage phenotype by ↑IFN-γ and *↓*IL-4 expression	mice	[169]
miR-222	Adipocyte	IRS-1	Liver	Promote insulin resistance	mice	[184]
miR-200a	Adipocyte	TSC1/mTOR	Heart	Cardiac hypertrophy related to diabetes	mice	[162]
miR-146b	Adipose tissue	Downregulate IRS-1 and GLUT4	Adipocyte	Promote insulin resistance	mice	[167]
miR-16, miR-27a, miR146b, miR-222	Hypertrophic adipocytes (VAT)	Upregulate GPAT-3 and DGAT-2; upregulate FSP27 and caveolin-1	Small adipocytes	↑Adipocyte lipogenesis (TAG synthesis) and lipid droplet formation ↓ WAT beigeing	rat	[157]
miR-155	ATMφ (M1)	Suppress PPARγ, GLUT4, and SIRT-1	VAT, muscle, and liver BAT and WAT	↑Insulin resistance and glucose intolerance *↓* BAT/beige WAT thermogenesis	mice	[181,182]
miR-486	ADSC	SMAD1	Podocyte	Diabetic nephropathy	mice	[175]
miR-199a miR 181a miR-495 miR-375	Liver	Suppress ER HSP70 chaperone BiP/GRP78 and IRE1α and ATF6 mRNA expression	Liver	Protect ER-stressed hepatocytes against apoptosis and alters adaptive UPR Promote peroxide-induced senescence	mice	[174, 176]
miR-30c miR-665 miR-34c	Adipocyte	Downregulate XBP-1 expression	Adipose tissue	Promotes adipocyte differentiation and alters ER adaptive UPR	mice	[174, 177, 178]
miR-23a-5p miR-33a-5p	Hepatocyte, adipose tissue	Inhibit ABCA1/ABCG1 reverse cholesterol transporter into HDL	Macrophage	Promote atherosclerosis and foam cell formation; *↓* HDL, NAFLD	mice, human	[164] [170]
miR-122	Hepatocyte	Activate TLR-9 via TNF-α	Endothelial cell	Angiogenesis and liver damage in NASH	mice	[173]
miR-192	Hepatocyte	Activate TLR-9 via TNF-α M1-like Mφ polarization	Endothelial cell Macrophages	Angiogenesis and liver damage in NASH NASH development	mice	[173] [171]
miR-128-3p	Hepatocyte	Inhibit PPARy	Hepatic stellate cell	Mediate hepatic stellate cell profibrogenic activation, NASH, and liver fibrosis	mice	[172]
miR-199a-5p	Hepatocyte	Suppress SIRT-1 and GLUT4	Pancreatic β cells	Reduced cell viability and promoted apoptosis and ROS formation in INS-1 cells in hyperglycemia	mice	[179]
MALAT1 ncRNA	Adipocyte	miR-181b and miR144/mTOR	Hypothalamus	Increased appetite and weight	mice	[180]

**Table 2:** A schematic summary of macrophage polarization. A2R, adenosine receptor 2; ACOD1, aconitate decarboxylase 1; AP-1, activator protein 1; ARG1, arginase 1; CARKL, carbohydrate kinase-like; IC, immunocomplexes; IDO, indoleamine dioxygenase; iNOS, inducible nitric oxide synthase; GATA3, GATA-binding protein 3; GS, glutamine synthetase; HIF1α, hypoxia-inducible factor 1-alpha; IFN-γ, interferon gamma; IL-, interleukin; IRF, interferon regulatory factor; MHC-II, major histocompatibility complex class 2; NF-κB, nuclear factor kappa-light-chain-enhancer of activated B cells; PPARγ, peroxisome proliferator-activated receptor gamma; SOCS1, suppressor of cytokine signaling 1; STAT, signal transducer and activator of transcription; TNF-α, tumor necrosis factor alpha; TGF-β, transforming growth factor beta; TLR, Toll-like receptor; VEGF, vascular endothelial growth factor. Reproduced from Viola et al., 2019 [211].

Mφ Polarization	Stimuli	Released Cytokines	Surface Markers	Metabolic Enzymes	Transcription Factors	Functions
**M1**	LPS + IFN-γ	TNF-α, IL-1β, IL6, IL-12, IL-23	CD80, CD86, CIITA, MHC-II	iNOS, PFKFB3, PKM2, ACOD1	NF-κB (p65), STAT1, STAT3, IRF-4, HIF1 α, AP1	Bacterial killing, tumor resistance, Th1 response, Glycolysis + ROS formation
**M2a**	IL-4/IL-13	IL-10, TGF- β	CD206, CD36, IL1Ra, CD163	ARG1, CARKL	STAT6, GATA3, SOCS1, PPARγ	Anti-inflammatory response, tissue remodelling, wound healing, urea cycle
**M2b**	IC, TLR Ligands/IL-1Ra	IL-10, IL-1β, IL-6, TNF-α	CD86, MHC-II	ARG1, CARKL	STAT3, IRF4, NF-κB (p50)	Tumor progression, immunoregulation, Th2 response
**M2c**	Glucocorticoids/IL-10	IL-10, TGF- β	CD163, TLR1, TLR8	ARG1, GS	STAT3, STAT6, IRF4, NF-κB (p50)	Phagocytosis of apoptotic bodies, tissue remodelling, immunosuppression
**M2d (TAM)**	TLR ligands + A2R/IL-6	IL-10, VEGF	CD206, CD204, CD163	ARG1, IDO	STAT1, IRF3, NF-κB (p50)	Angiogenesis, tumor progression
